# Theoretical and Data-Driven Approaches for Biomolecular Condensates

**DOI:** 10.1021/acs.chemrev.2c00586

**Published:** 2023-05-12

**Authors:** Kadi L. Saar, Daoyuan Qian, Lydia L. Good, Alexey S. Morgunov, Rosana Collepardo-Guevara, Robert B. Best, Tuomas P. J. Knowles

**Affiliations:** † Yusuf Hamied Department of Chemistry, University of Cambridge, Cambridge CB2 1EW, United Kingdom; # Transition Bio Ltd., Cambridge, United Kingdom; ‡ Laboratory of Chemical Physics, National Institute of Diabetes and Digestive and Kidney Diseases, National Institutes of Health, Bethesda, Maryland 20892, United States; ¶ Department of Genetics, University of Cambridge, Cambridge CB2 3EH, United Kingdom; § Cavendish Laboratory, Department of Physics, University of Cambridge, Cambridge CB3 0HE, United Kingdom

## Abstract

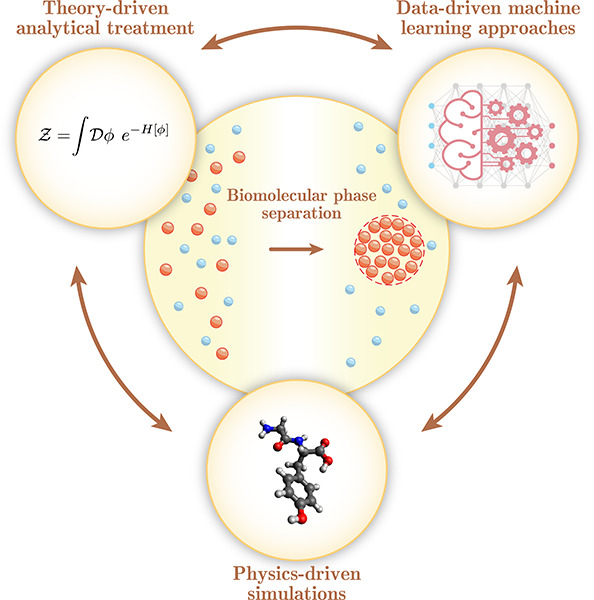

Biomolecular condensation processes are increasingly recognized as a fundamental mechanism that living cells use to organize biomolecules in time and space. These processes can lead to the formation of membraneless organelles that enable cells to perform distinct biochemical processes in controlled local environments, thereby supplying them with an additional degree of spatial control relative to that achieved by membrane-bound organelles. This fundamental importance of biomolecular condensation has motivated a quest to discover and understand the molecular mechanisms and determinants that drive and control this process. Within this molecular viewpoint, computational methods can provide a unique angle to studying biomolecular condensation processes by contributing the resolution and scale that are challenging to reach with experimental techniques alone. In this Review, we focus on three types of *dry-lab* approaches: theoretical methods, physics-driven simulations and data-driven machine learning methods. We review recent progress in using these tools for probing biomolecular condensation across all three fields and outline the key advantages and limitations of each of the approaches. We further discuss some of the key outstanding challenges that we foresee the community addressing next in order to develop a more complete picture of the molecular driving forces behind biomolecular condensation processes and their biological roles in health and disease.

## 1 Introduction

Phase separation is a fundamental phenomenon of many biological molecules, such as proteins and RNA, characterized through the demixing of a homogeneous solution into a polymer-rich and a polymer-depleted phase. Recent studies have highlighted that inside living cells, phase separation processes can lead to formation of biomolecular condensates that provide cells with the means to spatially and temporally organize their contents beyond what is achievable with just membrane-bound compartments.

Biomolecular condensates formed through phase separation processes have been found to play functionally important roles in a variety of biological pathways.^1,2^ Some of the best-studied examples of functional condensates include nuclear condensates such as nucleoli, which are the site of ribosome biogenesis,^3,4^ and cytoplasmic stress granules, which play a role in the cellular stress response.^5,6^ In multiple instances, biomolecular condensates have also been found to regulate gene expression^7,8^ and to facilitate cellular signaling pathways.^9−12^ Condensates are important for their contributions to healthy cellular function, but also because of associations between condensate dysfunction and disease.^13^ Disruptions to condensate formation have been linked to a number of diseases, most notably neurodegeneration^14^ and multiple forms of cancer.^6,15^

As the importance of biomolecular condensates is increasingly recognized and their varied roles in human health and disease are identified, it is crucial to develop approaches that can help us understand the factors governing separation phenomena that drive condensate formation. Insights both at the molecular level and in the context of the cell will be required to cellular condensates’ contributions to healthy function and to disease.

**Figure 1:**
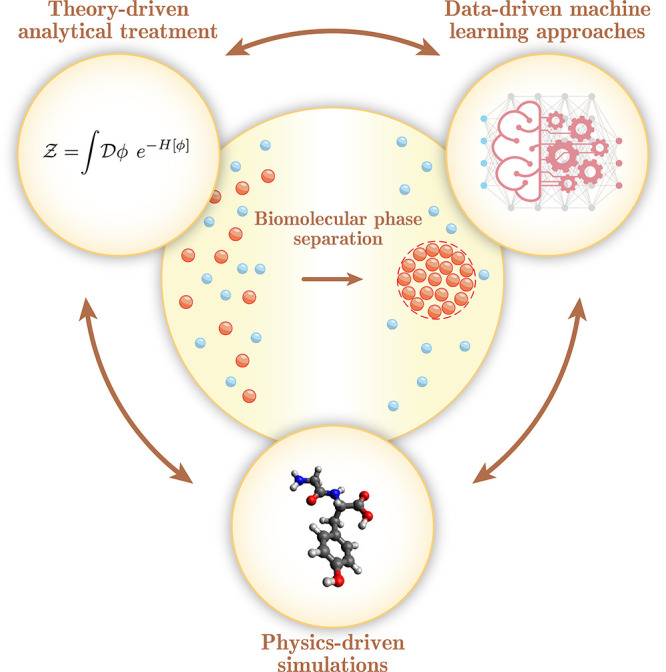
Theory, physics-driven simulations and data-driven machine learning approaches are all playing an important role in advancing our understanding of biomolecular condensation processes. This Review focuses on the recent advancements across all these three approaches outlining the advantages and limitations of each of the approaches. Simulation panel rendered with Avogadro.^16^

In this Review, we discuss three types of *dry-lab* approaches that have been used to study biomolecular phase separation processes, and how these methods can complement each other to collectively support the goal of better understanding the driving forces and the biological role of biomolecular condensation processes (Figure 1). We start by discussing the theory behind macromolecular phase separation and theoretical frameworks that have been developed for modeling macromolecular phase separation processes. We divide the latter frameworks into mean-field, molecule, sequence and residue level approaches according to the degree of coarse-graining they use. We proceed by reviewing simulations and molecular dynamics (MD) approaches and discuss how recent advancements in these areas have enabled their use for the examination of biomolecular phase separation processes, including in multicomponent systems. Given the large number of molecules that are required to simulate phase separating systems, we highlight the requirement for an appropriate balance between the detail that is used in simulations and its computational feasibility. Finally, we review how data-driven modeling and machine learning (ML) approaches have been used to predict protein properties and how these ideas have been extended to study protein phase behavior. In addition to reviewing the progress that has been made, we discuss the limitations of current methods and highlight the value of moving toward context specific predictions.

We note that while the different types of computational approaches each contribute unique insights, they also provide multiple interfaces for supporting and complementing each other. For instance, both simulations and machine learning models create computationally derived data that can be used to challenge and develop theories. Vice versa, the knowledge gained from theoretical approaches can enable the integration of appropriate inductive bias with machine learning models or with simulations. Focusing on the interface between MD and ML, ML can be used to increase the accuracy of MD force fields^17^ or to accelerate simulations and provide new routes to simulating systems over long time scales.^18^ In parallel, descriptors extracted from MD simulations can serve as inputs into ML models. Not only does this strategy lead to physically meaningful and interpretable inputs, it provides the possibility to capture how these properties vary in time.^19^

Importantly, alongside our review of the different types of computational approaches, we emphasize that the ability to perform well-characterized measurements on phase separation and phase separating systems is a key prerequisite for the validation of any new *dry-lab* approach. We are therefore looking forward to even stronger integration of computational models and experimental studies, including in an active learning format where computational models are used to guide experimental design, and attention is paid to studying systems in regimes where computational models appear most uncertain.^20−22^

## 2 Theoretical Approaches for Modeling Phase Separation

The physical concept of phases is formalized by assigning an order parameter ϕ(***x***) to each spatial coordinate ***x***. The definition of the order parameter varies greatly from system to system. For example, in a liquid–gas transition the order parameter can simply be the density of molecules or compressibility, while in random packing of spheres an ensemble of carefully constructed order parameters are needed.^23^ Despite their differences, order parameters serve a common aim of characterizing a physical state with a quantifiable metric, so that a mathematical framework can be developed to study transitions between states. Once a ϕ(***x***) has been defined in the system, typically an associated free energy density *f*(ϕ) is also constructed. A physical system evolves toward configurations that minimize the total free energy *F*[ϕ] ≡*∫*d***x****f*[ϕ(***x***)] and two important classes of ϕ exist: nonconserved and conserved ϕ. In the former case, ϕ can be changed locally to minimize the free energy without global constraint; classical examples include ferromagnetism^24,25^ and superconductivity,^26^ with order parameters being magnetism, and electron wave function, respectively. The most stable configuration is then simply determined by d*f*(ϕ)/dϕ = 0, and if multiple minima exist the one corresponding to the lowest free energy density will be the most stable state. In thermal equilibrium the system thus assumes a single value of ϕ throughout. By contrast, in biomolecular phase separation, the order parameter is the protein concentration and the total amount of protein has to be conserved in the whole system, leading to a globally constrained free energy minimization problem. Denoting the average protein concentration as φ̅ and total volume *V*, we then require ϕ(***x***) satisfies *V*ϕ̅ = ∫d***x***ϕ(***x***) = const. while minimizing *F*[ϕ]. As a result, the stability of the solution changes as φ̅ is changed and three regions can be identified: a mixed region with no phase separation, a metastable binodal region and an unstable spinodal region. The stability of a system can be probed by first partitioning it into separate compartments with different solute concentrations while preserving mass balance, and then calculating the total free energy change before and after the partitioning. If the free energy decreases for an arbitrarily small density difference then phase separation occurs spontaneously, since any density fluctuation could induce phase separation. This is the spinodal region. On the other hand, if the total free energy decreases only for large density fluctuations, the system is metastable and said to be in a binodal region. Boundaries of the binodal region correspond to globally stable configurations and mathematically solving for them gives rise to concepts of phase equilibrium,^27^ chemical potential μ and osmotic pressure Π. The μ and Π are both derived from the free energy density *f*(ϕ) so most equilibrium modeling attempts in protein phase separation focus on computing *f*(ϕ) and finding phase boundaries by matching μ and Π.^28^ In thermal equilibrium, the system is characterized by two regions having two different ϕ, and in each region ϕ is uniform except close to the interface. It is also possible to characterize proteins by proposing sequence-dependent parameters and investigating correlations between those parameters and phase separation propensity.

**Figure 2:**
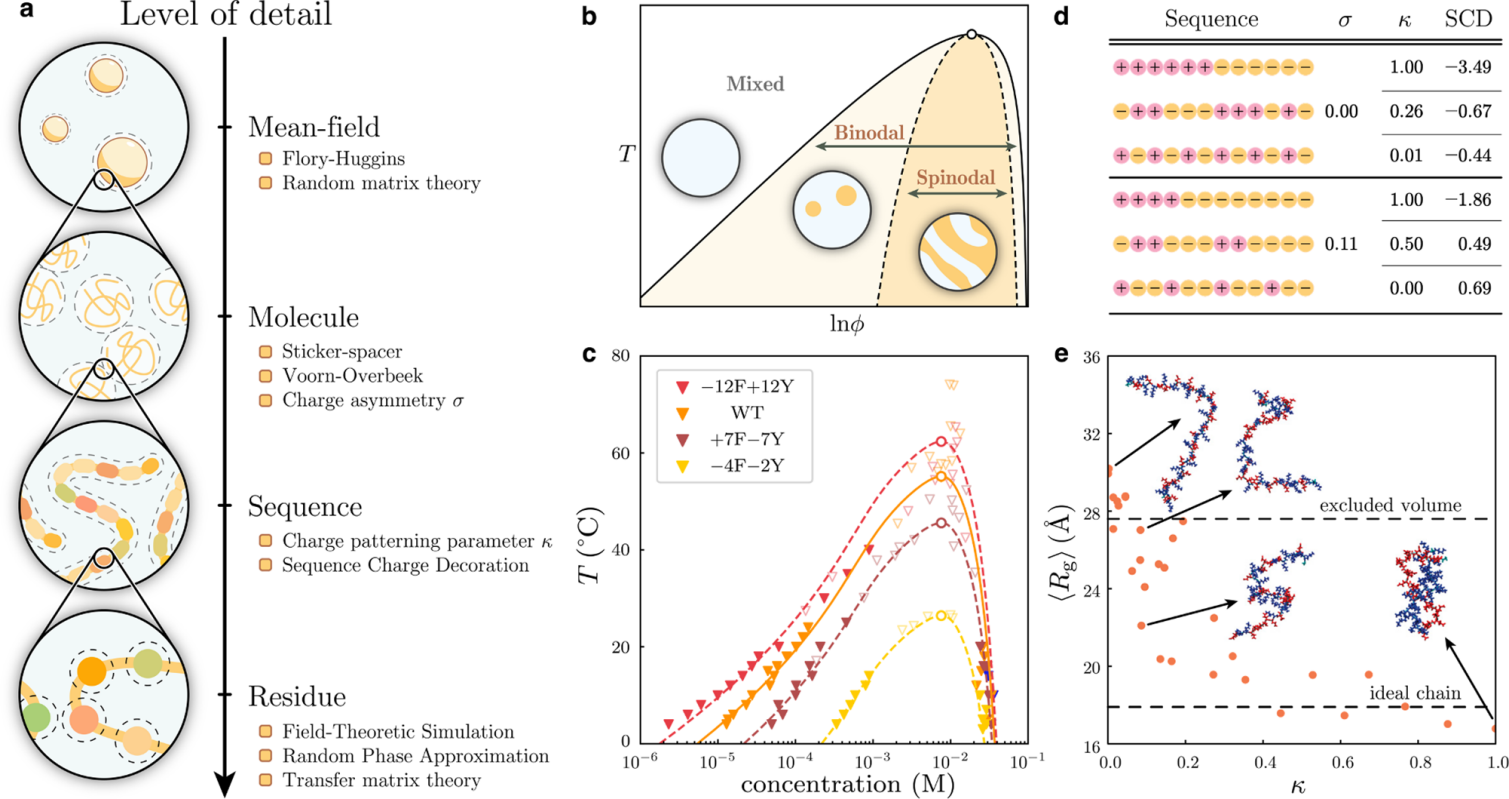
a. Summary of theoretical approaches reviewed here, arranged according to the level of physical detail considered. b. Illustration of the two-component Flory–Huggins phase diagram. At each temperature *T* below the critical point (hollow circle), three regions can be identified according to the total solute concentration ϕ: mixed, binodal and spinodal. Insets show characteristic appearances of the system in each region. c. Fitting of experimentally measured binodal concentrations of different hnRNPA1-LCD variants using the self-consistent solution. Adapted from ref (29). Copyright 2022 American Chemical Society. d. Sequence-dependent parameters that aim to capture composition and residue arrangement information in a single number. Illustrated are 2 groups of 3 sample sequences of 12 residues in length, with sequences within each group having the same composition and σ but different charge arrangements and thus κ and SCD. Blob size is set to 3 for κ calculation. e. Correlation between the radius of gyration ⟨*R*_g_⟩ and κ. Adapted with permission from ref (30). Copyright 2013 National Academy of Sciences.

Many approaches to modeling biomolecular phase separation exist. Here we discuss these approaches according to their degree of coarse-graining (Figure 2a): at the *Mean-field* level, interactions between proteins, nonprotein solutes and the solvent are phenomenological, examples include the basic Flory–Huggins theory, random matrix theory and active phase separation; at the *molecular* level, the composition of the polymer is taken into account, with the sticker-spacer model and the Voorn–Overbeek model focusing on computing *f*(ϕ) and the charge asymmetry parameter σ describing generic polymeric behaviors; at the *Sequence* level, the order in which monomers are arranged in the polymer is further taken into consideration, with relevant parameters including the charge patterning parameter κ and the Sequence Charge Decoration (SCD) parameter; finally at the *Residue*, level calculations of *f*(ϕ) are performed from first-principles using either a particle-to-field transformation as is done in Field Theoretic Simulation (FTS) and Random Phase Approximation (RPA) or a test-polymer thought experiment, leading to applications of the transfer matrix theory. A case can be made for all of the aforementioned approaches, while it remained an open question as to what degree of coarse-graining is most relevant for physiological systems and what should be expected of theories in the first place.

### 2.1 Mean-Field Theories Predict Rich Phenomenology

The Flory–Huggins formalism^31−33^ captures the basic phenomenology of density transitions and is the earliest attempt at modeling phase separation of polymer solutions. In this picture, phase separation is driven by a competition between interaction free energy and entropic free energy. In the simple binary case with ϕ denoting the polymer volume fraction, the total free energy density is given by
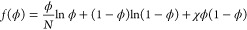
1where *N* is the polymer chain length and χ the effective polymer–solvent interaction parameter. Surface tension can also be added as a |∇ϕ|^2^ term to the above formulation that disfavors density fluctuations.^34,35^ Although simplistic, this free energy form predicts the core phenomenology, namely spinodal decomposition and binodal phase separation (Figure 2b). The spinodal concentrations can be worked out analytically^33^ while a system of transcendental equations needs to be solved for binodal concentrations. As a result, analytical solution for binodal equations does not exist and computation of the binodal concentrations have been performed largely through numerical methods in the past^28,36^ and only recently a self-consistent analytical solution has been derived.^?^ In the simple case of unit solute length *N* = 1, the dilute phase binodal concentration φ_dil_^bin^ can be obtained as
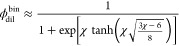
and for large χ the scaling law ϕ_dil_^bin^ ≈ *e*^–χ^ can be obtained. The expression for general *N* is more complicated, but a similar scaling law can be derived, giving ϕ_dil_^bin^ ≈ *e*^–*χN*^. The exponential dependence of the concentration on interaction energy and chain length explains why phase separation can be observed at concentrations spanning orders of magnitude differences. In contrast, the spinodal concentration ϕ_dil_^spi^ has a polynomial scaling law ϕ_dil_^spi^ ≈ 1/2*χN*; these boundaries are thus qualitatively different. Furthermore, the self-consistent iteration approach provides an efficient way of fitting experimentally measured boundaries to extract interaction energies (Figure 2c). An interesting result from the fitting above is that the effective polymer size is approximately 1.16 times the number of residues, reflecting the fact that one residue is slightly larger than the lattice size that is in turn determined by the correlation length of water molecules. It is straightforward to extend the theory to include more solute species^34,37^ while exploration of the high-dimensional phase space still has to be done numerically.

Difficulty in determining accurately the binodal concentrations has also resulted in efforts exploring the use of the spinodal as a proxy to study multicomponent systems.^38,39^ Diagonalizing the Hessian matrix of the free energy allows qualitative interpretation of experimentally measured tie-lines,^38^ and for a large number of solutes this path led to the application of random matrix theory in phase separation.^40,41^ This is especially pertinent considering the large number of protein species in living cells.^42^ The central idea is to treat the pairwise interaction parameter χ as a 2-dimensional matrix with each entry drawn randomly from a fixed underlying distribution, with the rank corresponding to the total number of solute species *s*. In the limit of an infinite number of solute species *s* → *∞* the statistical properties of the system become predictable. This led to 2 distinct modes of phase separation: a “condensation” mode with all solutes colocalizing in a dense phase and ∼*s*/2 “demixing” modes that preferentially include and exclude some of the solutes.^41,43^ The latter scaling is a direct result from Wigner’s semicircle law^40^ where ∼*s*/2 eigenvalues of the random matrix are smaller than 0, and the former arises from a distribution of interaction parameters with a nonzero mean. In comparison, the maximum number of phases that can coexist is ∼*s*, given by the Gibbs phase rule,^44^ and numerical investigation has shown that both scalings can be reproduced with a suitable choice of interaction parameter distribution.^39,45,46^ It still remains to be shown, however, that predictions from random matrix theory are physically and physiologically relevant.

The models presented so far only consider phase-separating systems at thermal equilibrium, while an important aspect of condensate formation in cells is the presence of energy-consuming cellular activities including post-translational modification (PTM) and cytoplasmic streaming.^47^ Two classes of activities can be identified: molecular modifications and intrinsic motility. Molecular modifications can include PTM, chaperone actions or artificial activation of protein constructs.^47−50^ In such systems, one can model different protein species as distinct types of solutes and use the Cahn–Hilliard framework of molecular fluxes^34,35,51^ to study the evolution of dilute and dense phases in time and space,^52−54^ and the activity is incorporated via introducing dynamic processes between solutes. Very generally, denoting different solute concentrations as ϕ_*i*_(***x***) with *i* an index for the solutes, and a generic free energy density *f*(ϕ_*i*_), the fluxes ***J***_*i*_(***x***) can be computed as  with *L*_*ij*_ the matrix of Onsager kinetic coefficients.^34,55,56^ The local concentrations ϕ_*i*_(***x***) then evolves simply via . On the other hand, the proteins themselves can acquire motility through ATP consumption using, for example, a motor.^57^ In this scenario, the activity becomes an intrinsic property of the solute and has to be modeled differently by either modifying the basic flux equation^58,59^ in a bottom-up manner or introducing a nonintegrable term to the free energy functional^60^ in a top-down manner. An interesting insight from the former approach is that given a density-dependent self-propulsion speed, phase separation arises from a jamming-like collective behavior even without any other interaction.^58^ The appropriate framework for modeling active phase separation thus depends largely on the underlying physics.

### 2.2 Molecular Details Correct for Composition Effects

The phenomenological nature of the χ parameter in the Flory–Huggins model motivates more detailed descriptions of its origin, especially since condensate compositions can be measured experimentally. As such, efforts to include explicitly electrostatic interaction and hydrophobic sticker-like interaction have been made over the past decades. The Voorn–Overbeek theory^61^ models electrostatic interaction arising from charged residues in the polymer by treating the solution as an ionic gas, from which results of the Debye–Hückel model^62^ can be used to give the electrostatic interaction energy *f*_VO_ ∝ *Q*^3/2^ with *Q* the total charge density. A comparison with experimental data gave mixed results,^63^ and discrepancies arise from intrachain correlations and charge density fluctuations that are not considered in the Voorn–Overbeek theory. Subsequent work to take polymeric properties into account showed that the charge-asymmetry parameter , where *q*_±_ is the fraction of positive/negative charges on a single chain, separates different polymeric configuration regimes^64^ and serves as a way to characterize a polymer based on its charge composition (Figure 2d). The σ represents the amount of uncompensated charges on a polymer chain and acts as a repulsive term. The full electrostatic interaction also includes an attractive term arising from charge fluctuations and the interplay among these two terms and the entropic free energy leads to different spatial conformations of the polymer. The relationship between σ and phase separation propensity was not studied in detail but the σ parameter still represents a novel way to characterize polymers.

Apart from electrostatic interactions, hydrophobic interaction also plays a large part in protein phase separation.^65−67^ The sticker-spacer model^68,69^ treats hydrophobic residues as generic stickers capable of forming strong, pairwise bonds, and the interaction energy arising from sticker bond formation can be computed by counting the total number of ways the stickers can be paired. When the amount of bonds exceeds a certain threshold a sol–gel transition is predicted^68,70^ where a gel is defined as a cluster of infinite size. When coupled with the normal Flory–Huggins free energy, which poses as weak, nonspecific interactions, the phenomenology of liquid–liquid phase separation and gelation can be rationalized under a common framework. These two energy scales—the strong sticker–sticker interaction and the weak aspecific interaction—appear to support recent findings that prenucleation clusters are observed at concentrations much higher than that predicted by the classical nucleation theory,^71^ and an interplay between phase separation and percolation was suggested.

### 2.3 Sequence Differences Lead to Distinct Behaviors

Interactions in the theoretical approaches presented above are determined by polymer composition but not the exact sequence in which residues are arranged in the chain. The sequence information, however, is an important aspect of protein interaction^66,72−75^ and are increasingly addressed. To explore differences between sequences that have the same overall composition but different arrangements, series of such charged sequences have been studied computationally^30,76,77^ and a charge patterning parameter κ was shown to correlate strongly with the polymer conformation state^30^ (Figure 2d, e). The κ builds onto the σ parameter by first subdividing a polymer into blobs and computing σ for each blob, and then calculating the variance of σ before normalizing it between 0 and 1. By construction, κ is smallest when the charges in the sequence are homogeneously mixed and largest when segregated, thus encoding the sequence information into a single parameter. The κ is, however, phenomenological in nature, and subsequently the Sequence Charge Decoration (SCD) was derived mathematically from polymer path integral,^77^ and it was further shown that effective interaction energy and binding affinity can both be computed from the SCD.^78^ The SCD is defined as  with *N* the total number of residues and *q*_*i*_ the charge at the *i*-th residue. The SCD is shown to correlate strongly with κ^77,79^ and serves a similar role in characterizing the degree of charge mixing in a polymer chain (Figure 2d).

### 2.4 Residue-Level Calculations Mimic Real Protein Systems

At the highest level of molecular detail are theories that aim to calculate *f*(ϕ) from first-principles analytically. Transfer matrix theory, most commonly used in optics and lattice field theories, has been shown to be also applicable in studying polymer solutions^80^ by drawing the parallel between time propagation and traversing along a polymer chain. The analysis starts by placing a “test polymer” in the solution and examining the monomer segment at the end of the chain. Different possibilities of the end segment environment are considered, where the segment can bind to a salt ion, a segment from a solute polymer or not bound to anything for example, and the corresponding Boltzmann factor assigned and organized in a column vector of size *h*, with *h* the total number of possible states. The theory then moves onto the next segment in the test polymer and the Boltzmann factors arising from possible configurations for the second segment are calculated and organized in an *h* by *h* matrix, because the energy for the second segment can be dependent on the first. This process is repeated in sequence until the end of the test chain and the partition function for the test polymer can be computed, from which the free energy is calculated analytically and phase equilibrium conditions can be solved numerically to find binodal concentrations. A drawback of this approach is that the theory is tractable for relatively simple polymers but *h* increases exponentially when polymers have more complicated sequences, and the resulting analytical form cannot be interpreted easily.

The alternative route, and one that represents the closest semblance to the physical system, is promotion of the particle picture to a field picture using the Hubbard–Stratonovich transformation^81,82^ and evaluating the partition function either via the saddle point approximation, commonly termed Random Phase Approximation (RPA) for historical reasons,^83,84^ or via complex Langevin sampling, termed Field-Theoretic Simulation (FTS).^72,85,86^ The exact energy of a configuration is first written down as a sum of all pairwise interactions and a density function is defined simply by assigning a Dirac delta at each particle location, allowing the interaction energy to be written in an integral form. Gaussian smearing of the Dirac delta is then performed before applying the Hubbard–Stratonovich transformation, resulting in a field representation of the partition function. This way, not only interparticle distances but also correlation effects due to polymer connection are taken into account. The resulting field integral, however, is hard to evaluate, and RPA is applied to approximate the integrand to a Gaussian form so that the integral can be carried out.^79,85,87^ RPA performs well in the dense phase, while large fluctuations in the dilute phase resulted in the breakdown of the Gaussian approximation, an issue that could only be addressed by numerical evaluation of the field integral via FTS.^72,85^ Although FTS represents a simulation method at first glance, the particle-to-field transformation makes the polymer density a simulation parameter and changing it does not affect the time complexity of each step, and simulations at concentrations spanning orders of magnitude can be performed on similar time scales. The FTS integrals investigated only consider electrostatic interactions and pairwise excluded volume interaction, with hydrophobic sticker interactions still missing in the picture. Extension of current models can potentially shed more insight into realistic protein solutions.

## 3 Simulations

Molecular simulations can add specificity to models of protein phase separation by describing in detail the structure and dynamics of biomolecules that emerge from physically motivated interaction potentials between their components. As biomolecular condensation has become a topic of interest, molecular simulation techniques and energy functions have been developed to study phase separation, especially among intrinsically disordered proteins (IDPs). By explicitly considering the individual components of a system and mathematically representing their interactions, simulations can resolve details of interaction dynamics among molecules, including residue-specific effects like those described in the stickers and spacers framework,^66,68^ and can describe systems of more complex composition, for example including multiple components and interactions among and between both folded and disordered domains, which would be much harder to do via analytical theory or machine learning approaches. Simulations can also provide insights into dynamical effects that would be harder to obtain by other methods.

### 3.1 Levels of Resolution

**Figure 3:**
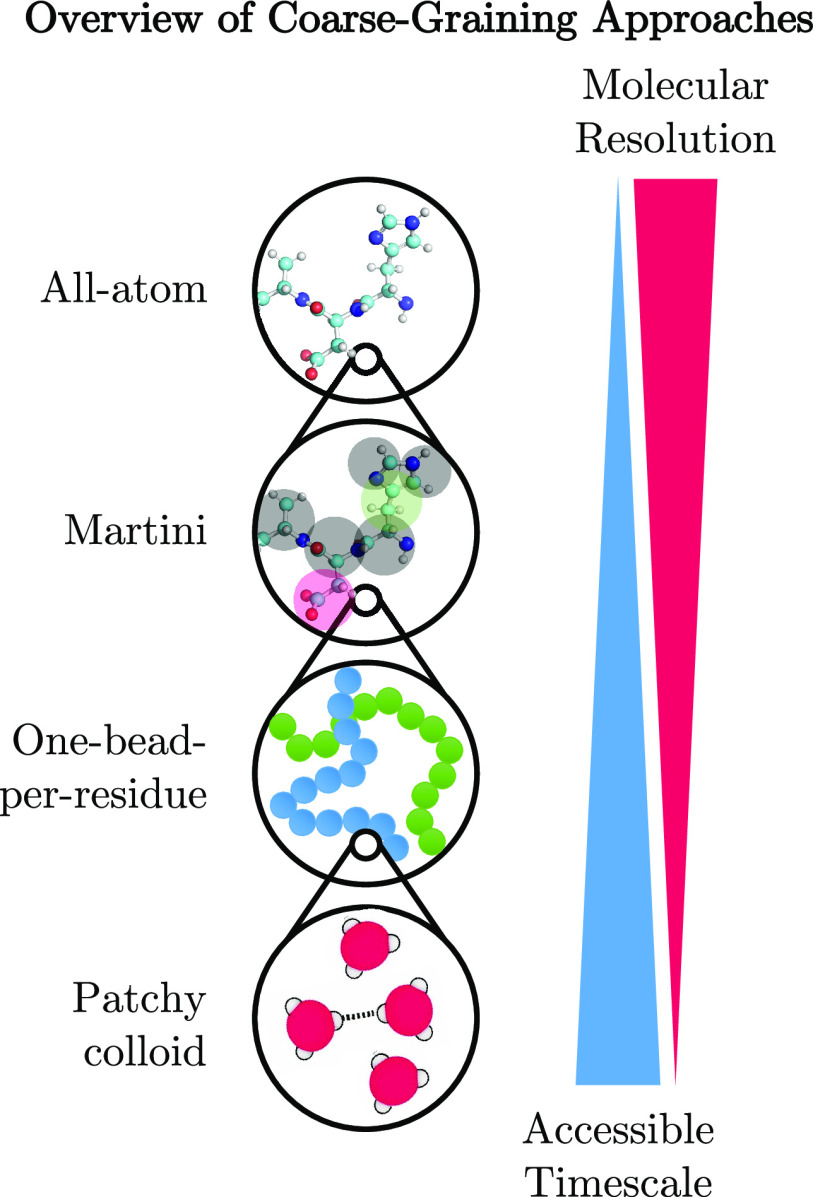
Summary of the range of molecular representations of varying resolution that have been used in simulations studies of phase separation. As the resolution decreases, the time scales accessible to these simulations become longer. The uses of each type of molecular representation are discussed below. Patchy colloid schematic adapted from ref (104). Copyright 2020 National Academy of Sciences.

Much like the theoretical approaches described above, simulations can represent biomolecular systems with a range of resolutions, and the details about a system that are accessible from a simulation depend on the resolution with which the system is represented (Figure 3). Such biological systems comprise many macromolecules, requiring significant computational resources to simulate, and this is especially true when studying the large numbers of biomolecules (on the order of hundreds at a minimum) required to form well-defined dense and dilute phases. Higher-resolution representations of the same system such as all-atom simulations have even more interacting bodies and are thus more computationally intensive to simulate. As a result, when considering simulation approaches, it is necessary to balance the detail used to represent a system with the feasibility of its simulation.

#### 3.1.1 Detailed Models Limit Simulation Length

The most accurate description of molecular systems is quantum mechanical, allowing for changes in chemical bonding. However, quantum chemical calculations are prohibitively expensive for large biological systems, usually being used only in cases where it is necessary to account for possible bond breakage or formation, such as in enzyme catalysis via a multiscale approach.^88^ So far, such effects have not been included in LLPS simulations, but they could potentially play a role in aging mechanisms; this might be most practically accomplished using empirically parametrized reactive potentials^89^ or force fields derived by machine learning.^90^

Without considering electronic degrees of freedom, the most detailed molecular simulations are so-called “all-atom” simulations in which all atoms in the system are represented as sites interacting via a pairwise potential, and held together by harmonic bonds within molecules; usually the surrounding solvent molecules and ions are also explicitly represented. By solving Newton’s equations of motion to determine the evolution of the system given the starting positions and velocities of all components, all-atom models provide detailed information about structural ensembles, dynamics, and intramolecular interactions within biomolecules. All-atom force fields have been refined over more than 40 years, and while they have historically been tested on and applied to study globular folded proteins,^91^ they are increasingly being adapted for IDPs.^92−95^ Using the all-atom CHARMM22* force field,^96^ Rauscher and Pomès simulated a collection of twenty-seven elastin proteins to confirm the liquid-like properties of its assemblies.^97^ However, because of the larger sizes of most phase separating systems when considered at atomic resolution, the duration of all-atom simulations of biomolecular condensates is limited on widely available computational resources. As a result, it is challenging to sample condensates for the time scales required to escape local caging effects that may lead to the appearance of subdiffusive dynamics, which is necessary to obtain reliable diffusion coefficients and other dynamical properties. Additionally, it is generally not feasible to determine the phase separation equilibrium using an all-atom representation from unbiased simulations because of the extremely slow escape of molecules from the protein-rich phase relative to accessible simulation time scales. Therefore, all-atom simulations of condensates thus far have started with a preformed condensed phase.

Despite these limitations, all-atom simulations still have a place in studies of biomolecular condensates. They have been used in combination with information from experiments and with coarser-grained simulations to provide detailed insights about specific interactions in systems where phase separation has been shown to occur. For instance, Zheng and colleagues performed microsecond-long all-atom simulations starting from various points in coarse-grained simulations of two different phase separating IDPs, which allowed them to track the diffusion of water and ions within and between the dense and dilute phases, and to identify the interactions contributing to IDP self-association in the dense phase.^98^ Atomistic simulations have also been used to generate starting conformations for coarser-grained simulations,^99^ to characterize the conformational ensembles of individual phase separating proteins and how they change with amino acid mutations,^100^ to simulate phase behavior of collections of single amino acids of different types,^101^ and to study condensate aging by zooming in on smaller, more specific structure forming sequence regions.^102,103^

All-atom simulations are also useful and reasonably efficient for calculating properties of isolated intrinsically disordered proteins such as approximate polymer scaling exponents, which are often found to be linked to their phase behavior; the second virial coefficient, which can be obtained from simulations of two proteins, is similarly useful although somewhat more challenging to compute accurately.^105^ Thus, predictions of single-chain properties from all-atom simulations under different conditions such as temperature or cosolvent concentration could, in principle, be used to predict the critical conditions for phase separation.

Properties of single chains or individual residues from all-atom simulations have also been used to parametrize coarser-grained models for a range of systems, including disordered proteins undergoing phase separation. Joseph et al. used all-atom representations of amino acids to calculate adjusted potentials of mean force between pairs of amino acids in the extreme salt conditions considered in their study, and to generate starting conformations for coarse-grained simulations of whole proteins.^99,106^ This approach is commonly used for coarse-graining at various scales, though it does risk importing biases in the finer-grained model into the coarser-grained model. For example, if the all-atom model favors excessive secondary structure formation or produces more collapsed conformational ensembles than the protein inhabits, then a resulting coarse-grained model could also oversample secondary structure contacts and collapsed conformations.^107^

Additionally, to reduce the number of calculations required without sacrificing atomic resolution in the biomolecules of interest, the solvent molecules in a biomolecular system can be replaced by a potential that implicitly accounts for protein–solvent interactions.^108^ One way of accomplishing this is by representing the protein’s solvation free energy with a combination of a mean-field interaction term and an electrostatic screening term as proposed by Lazaridis and Karplus^109^ and further developed by Vitalis and Pappu.^108^ The electrostatic screening can be tuned to reflect variations in the ionic strength of the solvent either by using explicit ions^108^ or via implicit screening. The latter approach is taken by Generalized Born models of implicit solvation; however, these have generally not been optimized for disordered proteins.^110^

#### 3.1.2 Coarse-Graining Makes Large Systems Accessible

Zooming out from the atomic level, coarse-grained biomolecular simulations represent multiple atoms via a single bead. The Martini force field is a widely used coarse-groaning approach that represents an average of four atoms in a single interaction site, each of which falls into one of four broad categories based on the chemical properties of its component atoms. The interactions between chemically bonded beads are derived from all-atom simulations, and interactions between nonbonded beads include Lennard-Jones and Coulombic interactions, which were optimized to capture experimental free energies of partitioning between the polar and apolar phases of chemical compounds. Reducing the number of interacting sites allows the model to retain some chemical specificity while running at least an order of magnitude faster than all-atom simulations on systems of the same size. Additionally, validation against a collection of properties of lipid bilayers showed that the Martini model could accurately determine the stress profile across a bilayer, represent pore formation, and could be used to calculate free energies of lipid desorption and flipping across the bilayer in agreement with all-atom simulations.^111^

**Figure 4:**
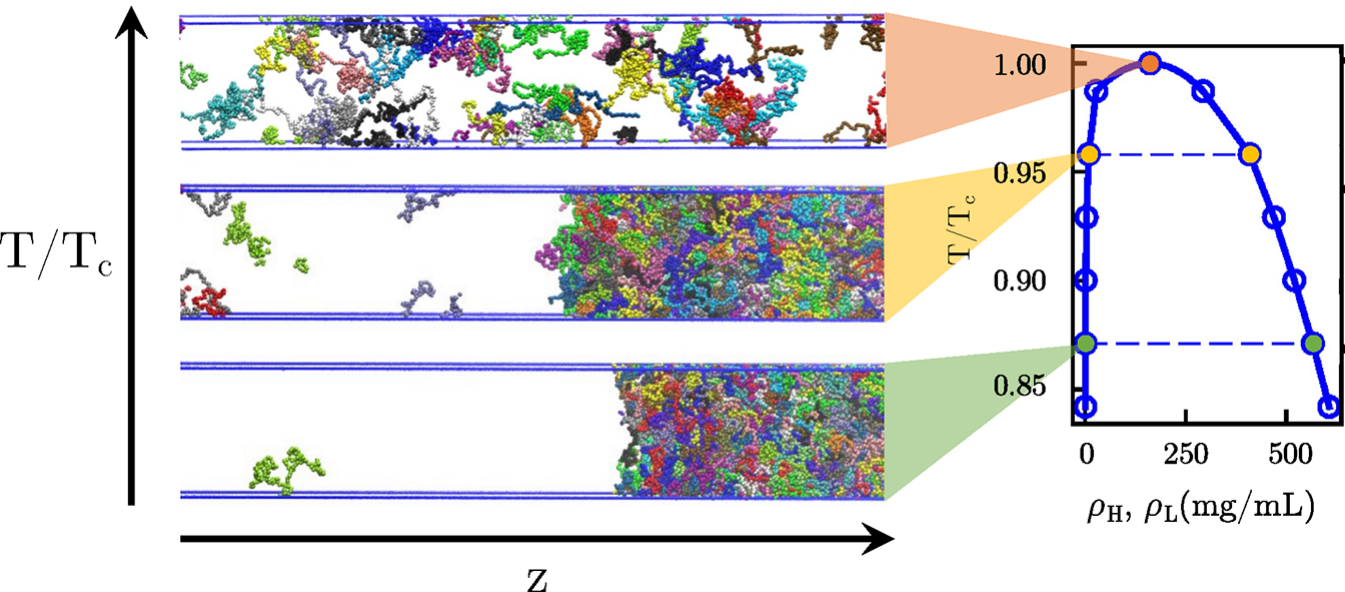
Direct coexistence simulations make connections to theory. By simulating a large number of proteins in a system, the densities of molecules in the dense and dilute phases under various conditions like those shown on the left can be used to construct a phase diagram for the system (as shown on the right). Figure adapted from Dignon et al.^112^

However, when applied to model proteins and other biomolecules, it became clear that the original Martini models’ intermolecular interactions were too strong.^113^ As a result, Martini simulations could not recapitulate observed behavior or produce values that agreed with experimental findings. For instance, osmotic second virial coefficients from Martini simulations could be off by a factor of 100,^114,115^ models of membrane-spanning proteins showed much more association and aggregation than structural and FRET data suggest,^116^ and simulations of carbohydrates also displayed excess aggregation that disagreed with light scattering measurements.^115^ These strong interactions also made it difficult to simulate protein phase separation using Martini, but recent adjustments to the model’s protein–protein interaction parameters to capture excess transfer free energies between the dense and dilute phases of protein condensates have made it possible to simulate phase separation of the FUS low-complexity domain.^117^ Similarly, comparison of the Martini 3 force field with global dimensions of disordered and multidomain proteins from small-angle X-ray scattering (SAXS) found the simulations were underestimating these proteins’ sizes, prompting reparameterization of the model by increasing the strength of protein–water interactions. The reparameterized model captures the larger dimensions of disordered and multidomain proteins, and agrees with global dimension and self-association measurements from SAXS and paramagnetic relaxation enhancement experiments.^118^ Both studies show that their adjustments to the force field make the Martini model more readily applicable to studies of phase separation.^117,118^ Additionally, systems including both disordered regions or flexible linkers and folded domains, as are common in multivalent proteins that phase separate to form a condensed phase, can also be handled in Martini by including elastic network or Go̅ interactions to define the folded domains. Remaining challenges include the development of an explicitly temperature dependent potential that would be required to reproduce temperature-dependent effects (e.g., the hydrophobic effect) in such a coarse-grained model.

**Figure 5:**
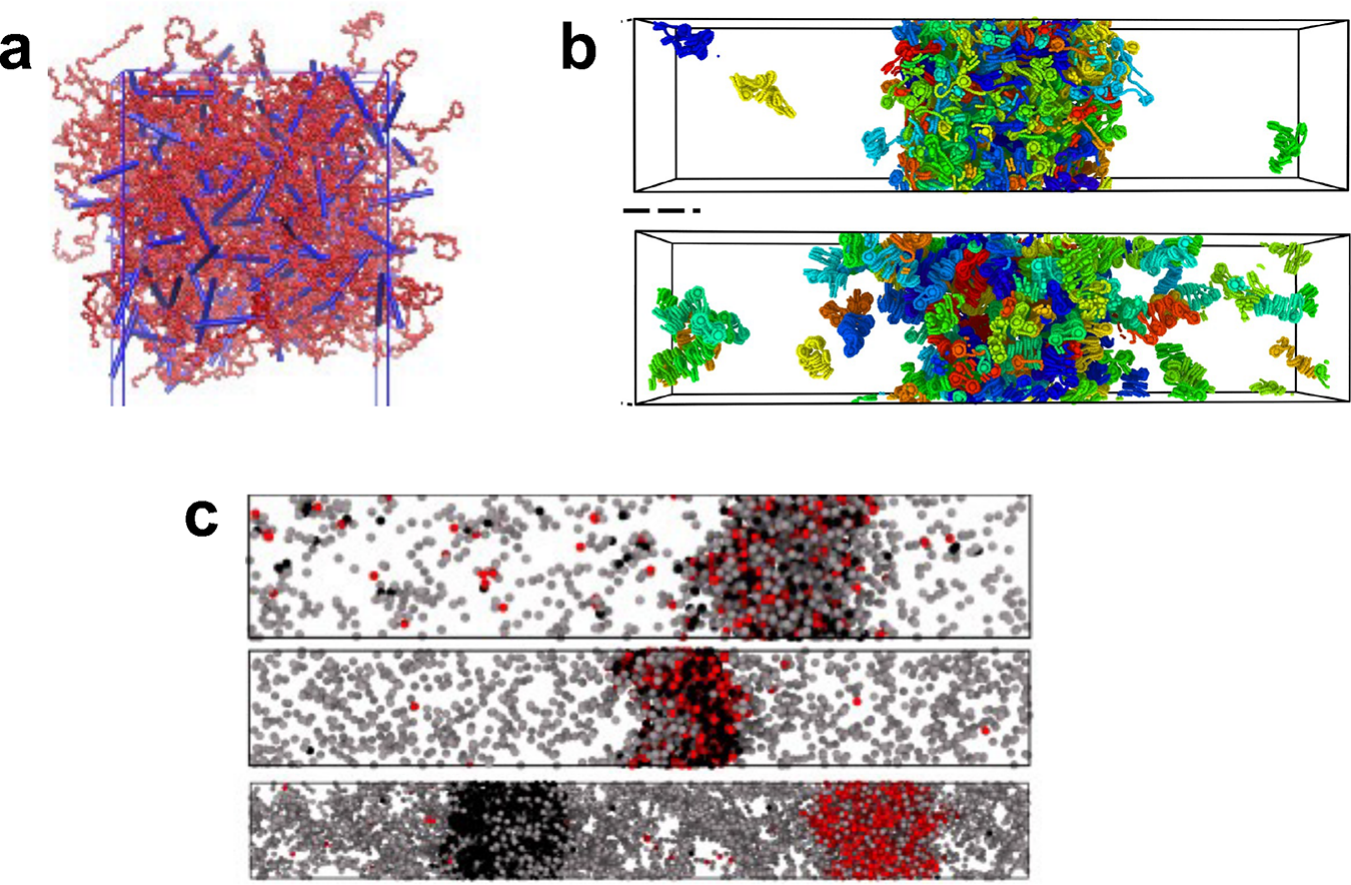
Applications of molecular simulations to phase separating systems. Panel A shows a coarse-grained model of the C-terminus of histone H1 (in red) condensing with DNA (in blue). Figure reproduced with permission from ref (125). Copyright 2022 American Chemical Society. In panel B, coarse-grained simulations were used to investigate the condensation of chromatin. Figure reproduced from ref (127). Panel C shows how patchy particle models can be used to investigate the effects of valence on molecular association and phase behavior, in this case on the structure and arrangement of molecules within the dense phase of phase separated systems. Figure reproduced from ref (132).

The most common class of models that have been employed for the study of disordered protein phase separation use one bead to represent each residue in a disordered protein chain, with electrostatic contributions and short-range interaction potentials used to describe the interactions between specific residue pairs. The first of these models was developed by Dignon and colleagues by combining a residue level coarse-grained protein model parametrized based on IDP radius of gyration data with a slab coexistence simulation approach that made it possible to converge system containing large numbers of molecules (Figure 4).^112^ This approach made it possible to determine critical temperatures and dense and dilute phase protein concentrations, which showed agreement with theoretical descriptions of polymer phase transitions from Flory–Huggins theory.^31−33^ Since the development of this coarse-graining framework, additional experimental data and atomistic simulations have been used to develop alternative residue-resolution coarse-grained models that aim to achieve quantitative agreement with experimental phase diagrams.^106,112,119−123^ The improved coarse-grained models can now quantitatively determine saturation concentrations^106,119,120^ and account for the effects of a range of temperatures and salt concentrations.^120^ These models have been used in a variety of investigations of phase separated systems, including to identify the interactions driving and sustaining phase separation of the FUS low-complexity domain,^124^ to understand salt-dependence in interactions driving phase separation,^99^ and to look at the effect of condensate aging on material properties and dense phase interactions.^102,103^ Coarse-grained models of nucleic acids have also made it possible to use simulations to look at protein–DNA interactions, including interactions between highly charged proteins and DNA in condensates,^125^ the interplay of H1, HP1 and DNA,^126^ to investigate the interactions driving phase separation of chromatin-associated proteins and its role in chromatin organization, and as part of a multiscale model of chromatin (Figure 5).^127^

Coarse-grained models also can be made more computationally efficient by confining molecular movements to a lattice as implemented in the LASSI model.^128^ However, this comes at the expense of information about chain dynamics that and transport properties such as diffusion and viscosity that can be gleaned from traditional molecular dynamics simulations.^102^

Despite their widespread use for direct coexistence simulations of phase separating IDPs, there is no single coarse-grained force field that represents well the interactions of both disordered and folded proteins in the same simulation. As many multivalent proteins with disordered regions or flexible linkers between folded domains are known to phase separate to form a condensed phase,^129^ representing heterogeneous systems involving both structured and disordered domains in a transferable, sequence-based model is an important challenge for accurate simulations of biomolecular condensates.

Beyond the sequence-based models described above, molecular simulations can also be used on a phenomenological level to test hypotheses about the principles governing a system’s behavior. One step simpler than the one bead per residue models, beads-on-a-string models lack information about residue chemical specificity and are instead composed of a small number of bead types with distinct interaction parameters (similar to early HP models for protein folding). These models have been used to investigate general properties of disordered protein phase separation, including the contributions of hydrophobic residues and their sequential arrangement to protein phase behavior, the influence of terminal residues on critical concentrations, and the interplay of traditional liquid–liquid phase separation with re-entrant phase behavior and aggregation.^130^ A similar model also showed that the sequence change decoration metric is helpful in distinguishing between proteins that will undergo phase separation versus aggregation, and that it is correlated well with critical densities for phase separation.^131^ This type of approach has also been applied to study aging, showing that the relative rates of condensate formation and the formation of strong interprotein interactions within a condensate affect the condensate’s transition to a more solid state and the shape of the resulting solid-like droplet.^103^

Moving away from modeling IDPs as flexible chains, patchy colloid models represent molecules as spheres that interact through specified attractive regions arranged on their surface. These models can be used to investigate the effect of valence on collective behaviors in solutions of molecules, including the role of valence in various aspects of phase separation of multicomponent systems, such as the role of network connectivity among scaffold and client proteins in the dense phase for condensate stability,^104^ and how differences in valency and binding affinity can promote layered organization of multicomponent condensates.^132^ Patchy particles have also been used to understand the mechanisms underlying size conservation in condensates with surfactant clients^133^ and the coexistence of multiple metastable droplets that do not immediately coalesce.^134^ Models with multiple distinct classes of particles with different valencies and interaction strengths have also been used to study how macromolecules modulate the phase separation of multivalent proteins by altering their critical concentrations^135,136^ and condensed phase material properties.^137^ Adding RNA to the system in the form of a long chain with multiple protein binding sites has also made it possible to interrogate the effect of relative RNA–protein and protein–protein interactions on the nucleation of phase separation, the stability of formed condensates and the internal distributions of molecules within droplets.^138^ Because of the fixed spherical shape of the molecules in these models, they are more appropriate for treating phase separation of multivalent folded proteins. It is naturally harder for such models to capture the role that flexible disordered regions of proteins play in promoting inter- and intramolecular interactions, although interactions between disordered proteins might be mapped to an equivalent “soft sphere” model.^139^

Building hypothesized constraints into a model makes it possible to test whether those constraints are sufficient to reproduce key experimental observations. These types of ground-up models are helpful for identifying the driving forces of a variety of processes, and have been used to study a range of processes from protein-driven membrane deformation and cell division^140,141^ to collagen self-assembly^142^ and the phase separation in many-component systems with a range of intermolecular interactions.^45^ While useful for focused investigations, these models are system-specific by nature, so they cannot be as readily applied to other types of systems.

### 3.2 Future Directions for Simulation Development

Molecular simulations’ explicit representations of biomolecules allow them to build on theory by accounting for hydrophobic and electrostatic interactions simultaneously and by incorporating residue specificity into inter- and intramolecular interactions. As reviewed above, there are a range of simulation approaches that have been applied to study biomolecular condensates. These simulations have been used both for explaining and adding detail to experimental findings about phase separation, and to gain new insights about condensate formation that can drive experimental investigations.

As the field advances, for simulations to continue to serve as useful tools to study biomolecular condensates, they need to expand to account for the wide variety of components of cellular condensates and the cellular environment. The biomolecular condensates found in cells, and even those studied by experimentalists *in vitro*, are often complex assemblies of proteins and nucleic acids, sometimes with post-translationally modified residues, and surrounded by solutions that can include ions, carbohydrates, molecular crowding agents like polyethylene glycol (PEG) and other biomolecules. To accurately represent these complex systems, compatible models for other molecules (nucleic acids, PEG, salt, sugars) with well-parametrized intermolecular interactions are needed. Coarse-grained models of DNA have been developed and used with coarse-grained protein models,^125^ and the Martini model was extended to model carbohydrates carbohydrates,^143^ but further development for these and other molecules is needed. To complicate matters further, cells are not at equilibrium like most simulated systems—although characterizing the equilibrium behavior is still a useful starting point for systems close to equilibrium. While it is feasible to simulate multiple distinct states of a system that is out of equilibrium, such as a protein before and after a post-translational modification, simulating active processes is much more challenging. An extreme example of an out of equilibrium system is the nucleolus, a condensate that is formed during ribosome synthesis, which might be characterized as a steady state in which production of ribosomes is balanced by creation of new ribosome components. In that case, a combination of equilibrium simulations with a kinetic or Markov state model would allow the complete process to be described. If developed, these additional components and capabilities would broaden applicability of molecular simulation approaches to more diverse biomolecular condensates and condensate-associated processes. In addition to allowing for simulation of more biologically relevant systems, simulations that can account for changes in ionic strength, pH and its effects on molecular ionization state, and even temperature can be used to probe the driving forces of phase behavior.^99^

Finally, an ongoing challenge for computational models of proteins, which also has implications for models of phase separation, is to parametrize transferable force fields that can accurately represent both structured and disordered regions in a single molecule without arbitrary scaling of interactions between disordered and structured regions. A model that can represent proteins with both folded domains and disordered linkers or tails would make it possible to further dissect the roles of structured and flexible domains in phase separation.^144^ Further, these models may also be used to consider a range of interaction specificities in a single system and dissect the role of specific, high-affinity interactions and lower-affinity associations to phase separation. Making coarse-grained models more representative of and compatible with details of the internal dynamics of condensates will make them more effective for studying processes that occur within the dense phase, such as the formation of internal structures during aging.

## 4 Machine Learning Approaches for Predicting Protein Phase Behavior

Machine learning and artificial intelligence have opened up new possibilities in many areas of life sciences and molecular biology.^145^ In this Section, we discuss the advancements that such data-driven modeling approaches have enabled in the context of analyzing proteins and their phase behavior propensity. We also review the limitations of currently used approaches as they are applied to the study of protein phase separation processes and highlight outstanding questions and challenges in the field, specifically around what concerns movement toward higher resolution and context-specific predictions.

### 4.1 Machine Learning Models Can Capture a Diverse Set of Properties

For a number of decades, the key quest of molecular and computational biology has been to understand how the sequence of a protein defines its structure and, by extension, its function. However, it was only recently that experimental techniques capable of acquiring protein sequence and function data have generated sufficient data to enable the effective introduction of data science approaches, such as machine learning, to address the key questions in the field of molecular biology and protein sciences. Many of the top performing approaches that have been developed over the recent years for predicting protein structure as well as other properties have relied on combining machine learning with some form of evolutionary information, typically expressed as multiple sequence alignments or position-specific scoring matrices that describe the sequence variations of the protein of interest across species. Early successes of this approach occurred can be found in the area of protein secondary structure prediction,^146,147^ and they were later followed by examples from a variety of other prediction tasks, such as predicting the subcellular localization of proteins,^148^ modeling protein binding pockets^149^ and designing proteins with desired function.^150^ Commonly recognized as the most notable milestone on the interface of computational sciences and molecular biology in the recent past was the application of deep learning with evolutionary information to predict protein structure from sequence, with AlphaFold^151^ demonstrating, as part of the Critical Assessment of Protein Structure (CASP) challenge,^152^ predictive accuracy comparable with the state-of-the-art experimental techniques.^153^ This achievement was soon followed by other approaches, such as RosettaFold,^154^ OpenFold,^155^ OmegaFold^156^ and ESM,^157^ often approximating or exceeding the performance of AlphaFold on the task of structure prediction.

**Figure 6:**
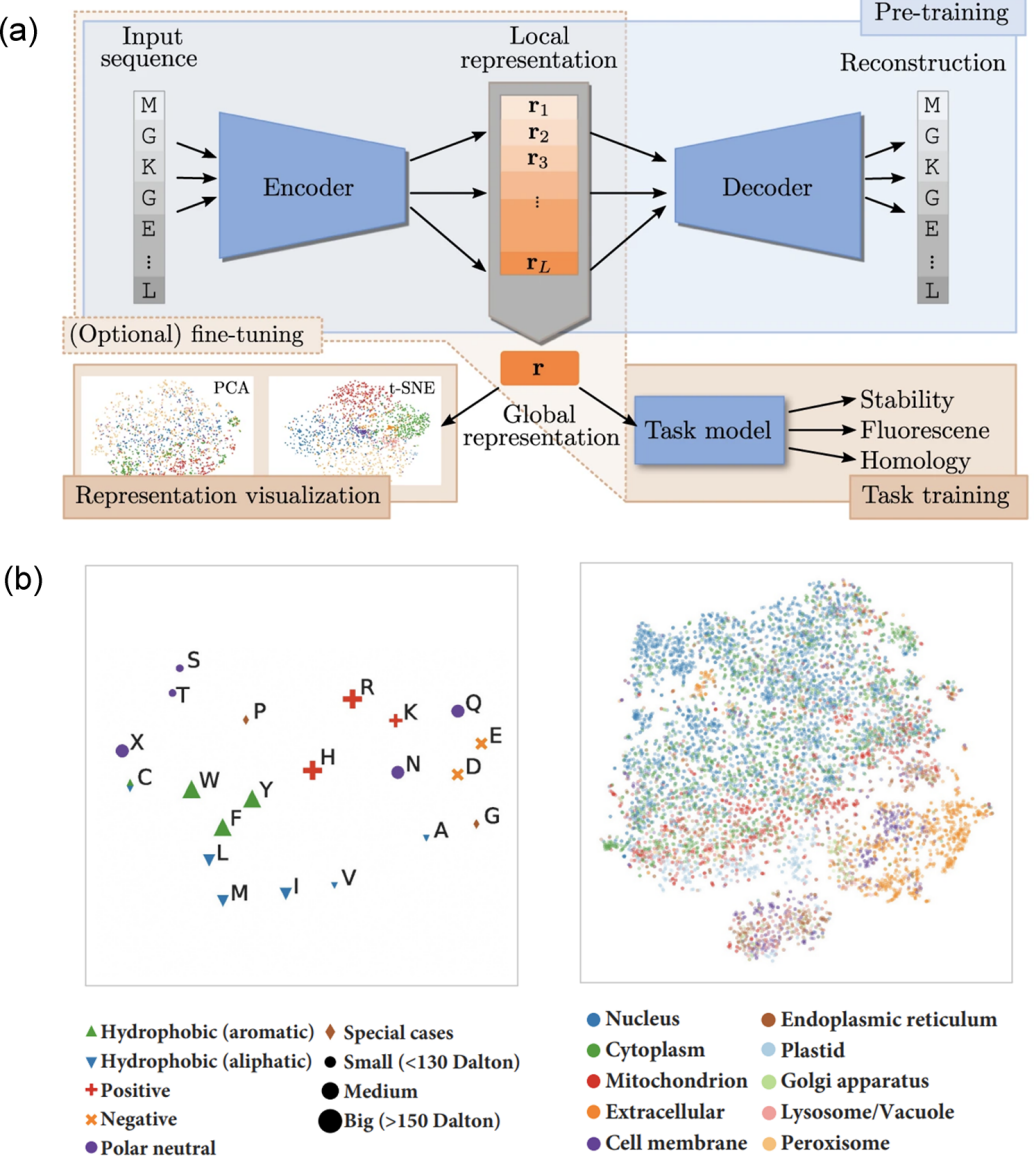
Protein language models (pLMs) can capture a multitude of protein properties. (a) pLMs are commonly trained on the largest protein reference databases by constructing a relevant self-supervised task to force the network to learn meaningful representations of protein sequences. A large variety of models exist with some based on the encoder-decoder architecture. Figure adapted from ref (158). The learned representations can be used as an alternative to hand-crafting features to train downstream models. (b) Low-dimensional visualization of the hidden state of Prot-Trans model.^159^ The representations learned cluster amino acids by their physicochemical properties (left) and by their subcellular location (right).

While powerful for prediction tasks with abundant labeled data, the use of models that rely on evolutionary information is not without its drawbacks. The drawbacks do not only pertain to the relatively expensive computational requirements for training an end-to-end model, but also to their limited applicability to proteins for which little evolutionary information is available. Therefore, an alternative set of approaches inspired by the rapid developments in the field of natural language processing (NLP) have opened a path toward learning protein properties without relying on evolutionary information as an explicit input. While the methods that rely on the development of protein-specific language models (pLMs) employ a diversity of network architectures and pretraining tasks, one common feature that they share is that they are trained in a self-supervised manner on large and diverse data sets of known protein sequences. During the training procedure, these models develop (or “learn”) high-dimensional representations of proteins, which can be used in a variety of ways (Figure 6a). For instance, their low-dimensional representations may allow direct extraction of biophysical information (Figure 6b, left) or they can be fed as input into another model trained on a specific prediction task with more labeled data (Figure 6a, bottom right).

The first NLP-inspired approaches that applied pretrained representations to protein property prediction were based on the principle of distributional semantics.^160^ The successes of this idea led to an explosion of interest in the field and was soon followed by pretrained models that involved more complex architectures, such as the mLSTM-based UniRep^161^ model and the ELMo-based SeqVec model.^162^ These more advanced techniques better captured the long-range information in protein sequences and allowed for learning protein representations that were more context specific. More recently, transformer-based architectures have been developed and they have been shown to outperform their predecessors on a number of tasks.^159,163−165^

It is worth emphasizing that the key feature of pLMs is that they output a learned representation of a protein sequence as a low-dimensional numerical vector. These representations have been shown to implicitly capture features relevant to the structural, spatial and functional organization of the proteome (Figure 6b), and are even being proposed for annotation of the full protein universe.^166^ This is in stark contrast to manually encoding protein properties as an explicit feature vector. Both are valid approaches to represent a protein sequence in a form suitable for machine learning and can be used as input to a downstream model trained on labeled data for a specific predictive task (Figure 6a, bottom right).

### 4.2 Curated Databases of Protein Phase Separation

**Figure 7:**
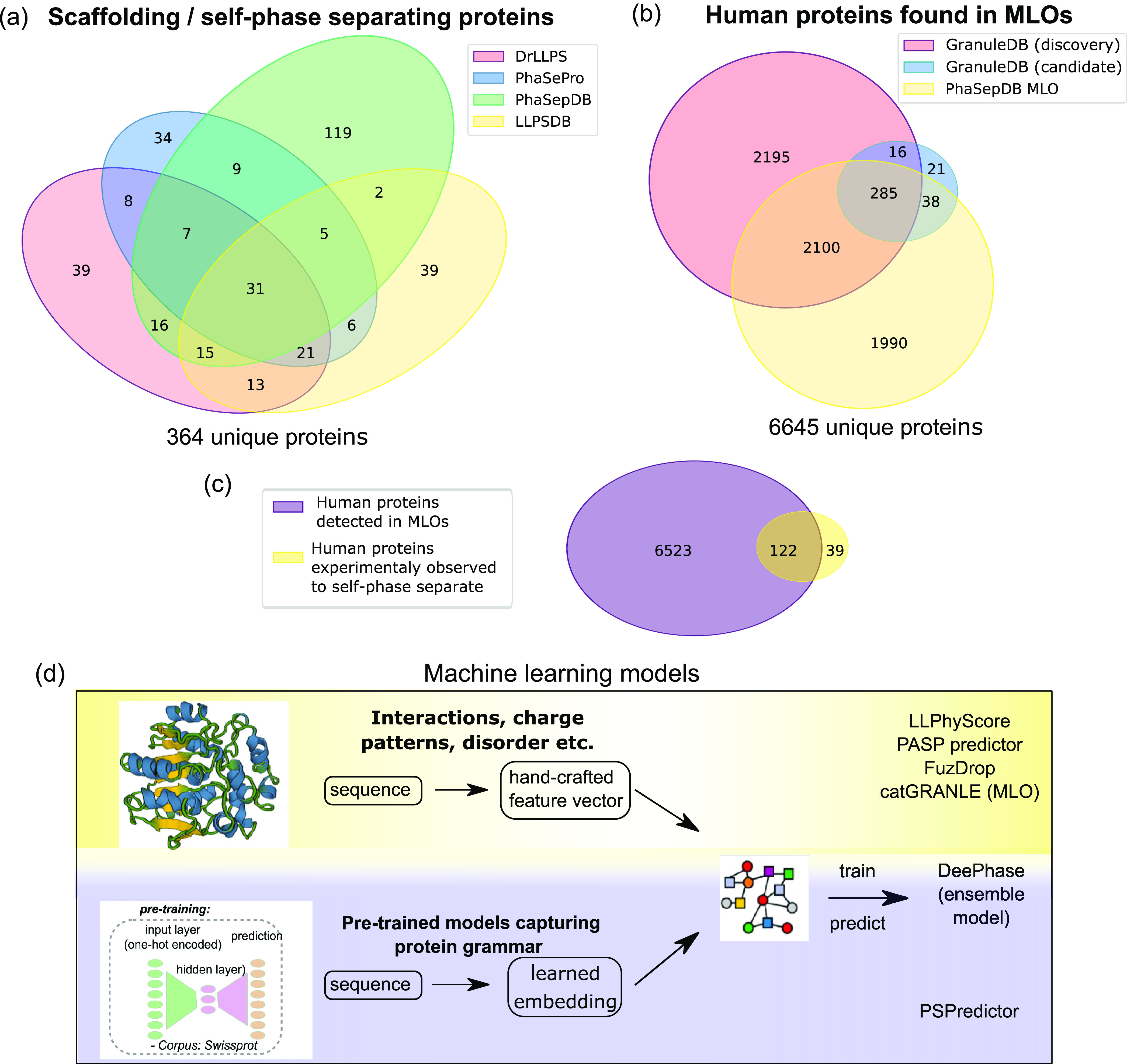
Data-driven modeling of protein phase behavior. Databases that characterize protein phase behavior can be divided into these that (a) highlight proteins that self-phase separate or act as scaffolds (four examples of such databases—PhaSePro, DrLLPS, LLPSDB and PhaSepDB—were considered; the values in the Venn diagram correspond to the number of proteins characterized by each database as self-phase separating or scaffolding) and (b) focus on protein localization into membraneless organelles (two examples, GranuleDB and PhaseSepDB, were considered; the values correspond the number of human proteins identified by each database). (c) The number of proteins from the human proteome that have been experimentally determined to undergo phase separation is a small fraction of the human proteome that has been found to localize into MLOs. (d) Based on existing data sets, a variety of machine learning approaches have been developed for predicting phase separation from the protein sequence. These can be divided into those that rely on individual knowledge-driven hand-crafted features (top), on nonexplicit protein featurisation from representation learning approaches (bottom) as highlighted in Figure 6, or on their ensemble (middle).

With investigations of biomolecular condensation processes becoming increasingly common across different laboratories, a variety of databases have been created to collate information on the phase behavior of different proteins. On the one hand are databases that specifically focus on gathering data from *in vitro* experiments. Such experiments present the possibility to understand in detail the molecular-level drivers of phase separation processes. The construction of these databases often relies on PubMed based keyword searches and while their broad focus overlaps, the details the databases provide differ. Here, we reviewed the data deposited in four of such databases (Figure 7a). First, the PhaSepDBdatabase^167^ describes proteins that can undergo phase separation and highlights if the sequence has been observed to do this on its own or not. It lists 356 entries across proteins with 203 unique Uniprot IDs where proteins have undergone phase separation on their own. Next, the LLPSDB database^168,169^ should be highlighted not only storing data on cases where proteins have phase separated but also doing this in conjunction with the experimental conditions under which their phase behavior has been probed. The database even includes a handful of phase diagrams collated from the literature. This feature gives the user the opportunity to use the information in a flexible manner to develop their own categorizations and annotations. Altogether, the database highlights 640 sequences that undergo phase separation with the sequences being protein constructs from 135 unique Uniprot IDs. Third, the PhaSePro database^170^ lists a relatively small set of manually curated LLPS-prone proteins (121 in total). It substantiates this information with the specific molecular interactions that are likely to drive the condensate formation and the involvement of and dependency on other components such as RNA and membranes and even the effect of post-translational modifications. Finally, the DrLLPS database^171^ stands out in the richness of its annotations. It aims to separate proteins into scaffolds (drive LLPS; 150 unique sequences), regulators (contribute to modulating LLPS; over 900 sequences) and clients (might be dispensable for formation of MLOs; over 8000 sequences). It further divides proteins into distinct condensate classes and outlines potential orthologs of these proteins across species. The database integrates information from a variety of other bioinformatic databases, so that the phase separation data would be accompanied by domain annotation, genetic variations, cancer mutations and drug-target relations.

**Figure 8:**
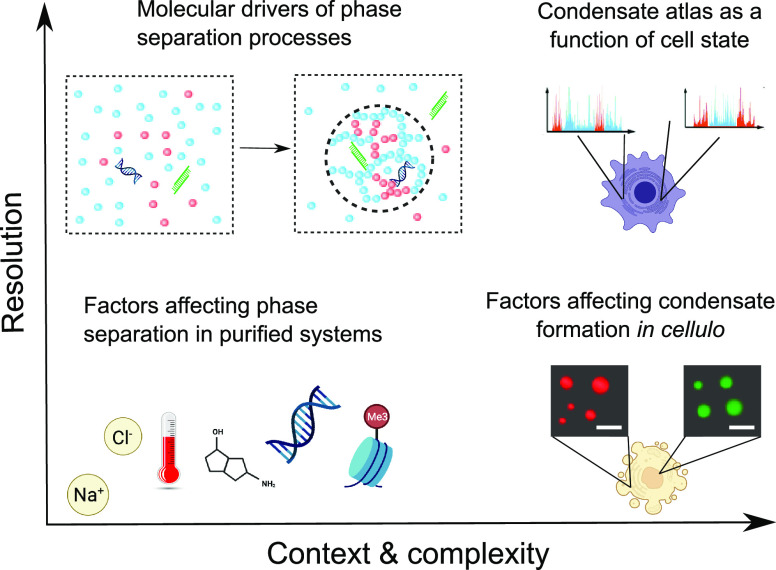
Capabilities of current predictive algorithms related phase separation processes can be advanced by accounting for the surrounding environment and its complexity to make the models context-specific (*x*-axis) as well as by understanding the molecular-scale drivers of the processes to increase the resolution of information that is obtained (*y*-axis).

On the other hand lie approaches and databases that do not aim to highlight scaffolding or self-phase separating proteins but instead focus on capturing the full diversity of sequences that have been found in cellular biomolecular condensates or membraneless organelles (MLOs). As has been highlighted by a number of studies, protein localization into MLOs is not necessarily dependent on the protein undergoing a phase separation process on its own. Many proteins are likely incoorporated into condensate bodies via interactions with other biomolecules or cofactors. The PhaSePro database outlined above captures this aspect by collating data from experiments that have shown protein localization into MLOs either in a high-throughput discovery-based manner (e.g., organelle purification, proximity labeling and immunofluorescence image-based screening) or in a lower throughput manner via direct experimental validation (e.g., immunhistostraining).^167^ Another relevant resource for MLO composition is the RNA Granule database (GranuleDB). In contrast to the PhaseSepDB that collates data across 73 different MLOs, the GranuleDB specifically focuses on proteins found in stress granules and P-bodies^172^ (Figure 7b). However, it collates these data across a variety of different studies performed by using different cell lines and growth conditions or stressors. The GranuleDB similarly distinguishes between candidate-based cases (cell biological, physical association or genetic evidence for the components) and discovery based approaches (purification of the condensates or on proximity-dependent biotinylation techniques prior to a mass spectrometry-based characterization step). When focusing on the human proteome of these databases, we see a substantial overlap in the identified proteins with the majority of the proteins that are picked up by candidate-based approaches also identified in discovery based approaches as expected (Figure 8b). The substantial number of proteins captured by the PhaSepDB database but not by the GranuleDB database can be rationalized by the former database collating data across all different MLO bodies, rather than just stress granules and P-bodies. The high number of proteins that is part of the GranuleDB but not the PhaSepDB data set, however, is more surprising at the first sight but likely illustrates the degree of variation that the specific context (e.g., cell line or the stressor) introduces to the composition of the condensate body. Indeed, when focusing on the set of genes that had been identified by both databases, we can see that over half of these proteins have been observed in condensates by at least two experiments while within the set that does not overlap with the PhaSepDB data only about one-sixth has been seen in more than one study.

Altogether, the databases that characterize the proteomes of different MLO systems list over 6600 proteins that have been found to localize into MLO bodies. This is a very notable fraction (around one-third) of the total human proteome, highlighting the importance of biomolecular condensates as a cellular phenomenon. Noteworthily, however, only 122 of these proteins have been seen to self-phase separate even when combining data across all the four databases outlined above (Figure 7c).

### 4.3 Data-Driven Approaches to Predicting Protein Phase Behavior

As described above, the emerging interest around phase separation processes has resulted in a multitude of experiments being performed that would allow elucidating the driving forces of phase separation processes on different proteins. Such experiments have either introduced different types of mutations (truncations, deletions, insertions) to the wild type protein sequence or, alternatively, have generated sequences *de novo*.^173^ Cumulatively, the investigations have identified a number of sequence-derived features that are linked to an elevated propensity of a protein to undergo phase separation. For instance, experiments performed on the fused in sarcoma (FUS)-family proteins have highlighted that high abundance of arginine and tyrosine residues in prion-like domains anticorrelates strongly with the saturation concentration of the protein and, moreover, specifically shown that the latter value is inversely proportional to the product of the number of tyrosine and arginine residues in the sequence.^174^ These arginine and tyrosine residues can be regarded as sticker regions according to the stickers-and-spacers framework.^66^ Similarly, experiments with TAR DNA-binding protein 43 (TDP-43) have eluded the relative positioning of tryptophan and other aromatic amino acid residues within the sequence as a key factor that governs the phase behavior of this protein.^175^ Examination of LAF-1, a DDX3 RNA helicase found in P-granules inside *Caenorhabditis elegans*, has eluded electrostatic interactions within disordered regions and, specifically, the arginine- and glycine-rich domain of the N-terminal disordered region of LAF-1 to be a key feature for its condensate formation with this domain being both necessary and sufficient for the phase separation process to occur.^176^ Crucially, multivalent interactions and oligomerization of the monomeric form of a protein, are believed to drive the phase separation of UBQLN2^177^ and BAG2^178^ proteins. Similarly, the phase separation of G3BP1, a common stress granule protein, has been shown to be initialized by oligomerization, specifically the NTF2 domain.^179^

Building on these observations, a number frameworks have been established to estimate the propensity of a protein to undergo phase separation from its sequence. One of the earliest algorithms was PLAAC.^180^ It used Hidden-Markov-Models trained on a small set of protein sequences to identify prion like domains (PLDs) within proteins. Despite being trained on yeast data, the approach has been found to work well on the human proteome. Two other predictors, R+Y and DDX4-like, have focused on understanding the key interactions that drive the phase separation of the FET-family and DDX4-family proteins, respectively, and expanded these leanings across the full proteome to identify additional condensation-prone sequences. The key determining features for these two approaches were found to be the statistical frequency and spacing of arginine and lysine residues for the FET-family proteins^174^ and that of the arginine and phenalynine residues for the DDX4-family proteins.^181^

CatGRANULE^182^ was one of the first algorithms that, instead of identifying phase separation prone sequencing, aimed to allocate sequences a score. Relying on sequence composition statistics, specifically on the weighing of residues based on their predicted disorder, nucleic acid binding propensity, sequence length and the frequencies of arginine, glycine and phenalynine residues, the algorithm distinguished yeast proteins annotated as granule forming from the rest of the yeast proteome. Another statistical scoring algorithm, PScore, first used sequence composition to predict the availability of π-groups for forming planar contacts with other π-groups. It then used π contact predictions to distinguish known phase-separating proteins from proteins found in the protein database to estimate the phase separation propensity of each protein.^183^ The planar π–π contacts on which this predictor was based are associated with structural disorder, kinks in beta strands, nucleic acid binding, increased interactions with solvent, aromatic residues, and arginine. These attributes set good basis for the algorithm serving as a good proxy for identifying phase separation prone proteins.

Vernon and Forman-Kay recently reviewed the predictive power of these approaches focusing on the PLAAC algorithm, LARKS, R+Y, DDX4-like, catGRANULE and PScore and CRAPome approaches.^184^ They referred to these predictors as first-generation approaches as they exhibit a common feature of using just a single or a small set of knowledge-driven features to characterize how likely a protein is to undergo phase separation. Their systematic analysis showed that there is a common set of proteins captured correctly as phase separating by all of the predictors with RNA-binding proteins in particularly confidently predicted correctly. The investigation further revealed that proteins whose biological phase separation involved folded domains, either in multivalent interactions or via post-translational modifications, were classified incorrectly by all the predictors, clearly highlighting the limitations of these approaches.

The availability of databases that collate information across a variety of studies to describe the phase behavior of hundreds of different proteins as described in the previous section has recently opened up the possibility to use more complex data-heavy strategies for predicting biomolecular phase behavior. In contrast to the first generation approaches described above, these models always consider a variety of features at once and use available data to learn relationships between the designed features that best describe the available data.

A number of such predictive algorithms using this approach have been developed over the past few years^185−188^ as is outlined in Figure 7d. Two of the first models put forward shortly after the first databases were released were the DeePhase predictor developed by Saar and Morgunov et al.^185^ and the PSPredictor algorithm by Chu et al.^187^ PSPredictor was reliant on features derived from pLMs, specifically the word2vec model (Figure 7d, bottom). This step limited the interpretability of the PSPredictor approach. The DeePhase algorithm combined learned representations with knowledge-driven hand-crafted features (Figure 7d, middle) with the final model relying on creating and ensemble between these approaches. This step of combining two orthogonal featurisation strategies was taken to facilitate arrival to a robust outcome in what is considered a relatively low-data regime. There have also been a variety of models that have relied solely on hand-crafted features (Figure 7d, top). One of the approaches that specifically exploits the fact that the model was built on explicitly defined descriptors rather than learned embeddings for introducing model intepretability is the LLPhyScore algorithm.^188^ When developing the model, the authors carefully divided features and interactions into long- and short-range interactions. Through competitive feature training, their results suggested the relative importance of long-range interactions over short-range ones.

### 4.4 Open Challenges: Resolution and Context

The majority of the approaches reviewed in this Section have yielded as an outcome a score that described the propensity of the molecule to undergo a phase separation process. While useful as a high level descriptor, such a single value is inherently limited in its interpretability as phase separation processes are known to be highly context specific. In this Section we outline a handful approaches have emerged over the recent years that have specifically been designed to overcome this limitation dividing them into those that ensure that the predictions are more context specific or increase the resolution at which information could be obtained (Figure 8).

First, Farahi et al.^189^ used available phase separation databases (Section 4.2) to compile a list of 78 proteins for which they were able to quantify the minimum concentrations at which the proteins had been observed to phase separate *in vitro*. For the purpose of their analysis, they used the latter value as an estimate of the saturation concentration, *c*_sat_, of the protein. In parallel, they turned to the PaxDB database^190^ to retrieve cell- and tissue-specific abundance of these proteins. Combining these two resources, their approach identified some proteins for which tissue specific concentrations reached values similar to the approximated saturation concentrations *in vitro*. This included nucleolar proteins (NPM1) and FUS-family RBPs (HNRNPA1, FUS, TARDP). For the majority of cases, however, the experimentally determined *in vitro* saturation concentrations was higher than the estimated cellular concentration of the protein, often by 1–2 orders of magnitude. Given that many of the 78 proteins included in their have been observed not only to phase separate in a purified form but to also localize into MLOs in cells, this discrepancy is likely due to strong local variations in protein concentration across the cell, the important role that other cellular components play in the formation of membraneless organelles, or their combination.

To advance a step further, recently, an attempt was made to not only predict the saturation concentrating of a protein at specific conditions but also its phase diagram.^191^ The authors worked with a list of around 900 experiments performed on 366 unique sequences across 137 proteins and built a multihead neural attention-based deep learning model. The performance of the developed model in a classification task (predicting if phase separation is likely to occur at unseen concentration) remained limited, reaching an area under the receiver-operator characteristic curve (a measure of the usefulness of the model obtained by integrating the area under the curve highlighting the true positive rate (*y*-axis) and the false positive rate (*x*-axis) at all classification thresholds) of 0.64 in 5-fold cross-validation process, likely due to the sampling of the data points across a multidimensional space being sparse. However, the concept of transitioning from phase separation propensity to the prediction of the experimental conditions under which specific sequences are likely to undergo phase separation is to be acknowledged.

An additional dimension of context-specificity relates to the inclusion of additional components in the system. The data on how the inclusion of molecules, such as small drug-like components or RNA, has begun to emerge, but the volumes of these data have not made current data sets amenable to machine learning approaches. Specific approximations can, however, be made to propose new insights. For instance, Zhu et al.^192^ have highlighted the possibility to predict the effect of oligonucleotides on phase separation by building two different predictors: one that had been developed to predict phase separation *in vitro* and an additional model that predicted partitioning of proteins into reconstituted RNA-rich granules. By contrasting the two predictive models, the authors proposed a set of proteins that would have their phase separation propensity heavily enhanced in the presence of oligonucleotides. One of the predicted candidates, HMGA1, was experimentally demonstrated to follow this predicted trend.

Models that predict protein localization into condensates inside the complex cellular environment have been developed. Indeed, it should be noted that some of the first generation predictors described earlier, such as PLAAC and catGRANULE, were built on known components of MLOs rather than purified systems. As such, these models could be regarded as the first generation MLO composition predictors. More recently, Kuechler et al.^193,194^ gathered a comprehensive list of stress granule components across multiple studies and used this collated data set to examine how the properties of proteins, such as their amino acid composition or global physicochemical properties like solubility and melting temperature correlate with their localization into stress granules. Based on these data and descriptors, the authors subsequently developed a machine learning model that predicted the composition of stress granules (MaGS) and employed it to propose additional components that may be localizing into stress granules but had not been detected by current characterization protocols. Two of their proposed candidates, Zyxin and SynJ, were later validated to localize into stress granules upon arsenate stress, suggesting predictive models have the capability to unravel components of MLOs that are challenging to identify with hypothesis-free experimental approaches. A recent study by Chen at al. used the annotations in the PhaSepDB database to develop a machine learning model that predicted the localization of proteins into condensates from the protein sequence. As the training data for the model covered proteins from a variety of MLOs, the predictions were not specific to a single condensate system like the MaGS described previously, and the authors made limited attempts to understanding which of the high scoring components were likely to localize into the same condensate system. In conclusion, while these approaches have generated the possibility to investigate and model phase behavior in a complex cellular background, they are limited to a specific environment, such as the conditions inside the specific cell where the data was acquired and do not consider the cellular environment as an input to introduce context dependency.

In parallel, another direction toward which future approaches should aim to move is the resolution at which the systems are being characterized (Figure 8). ML approaches have so far made limited systematic attempts toward identifying the regions or domains of proteins that are relevant for protein separation. This is partly related to their inherent limited interpretability and it has been helpful to see certain recent predictors explicitly aiming to overcome this as described in the previous section.^188^ Crucially, these attempts have remained global without highlighting which aspects define the phase behavior of a specific protein sequence. Movement toward such a capability would permit understanding the effect of specific mutations on protein phase behavior. In the context of predicting phase behavior inside a cell, further resolution is desired to elucidate which components delocalize into the same condensate system. Current models that group different types of MLOs together into a single annotation are limited in achieving this. Experimental data from spatial proteomics studies, such as those acquired through fractination centrifugation^195^ or through imaging,^196^ can fill an important gap here and provide a route to subdividing condensation prone proteins into separate systems. Predicting such localization as a function of the cell state is likely to be one of the common goals driving further research in this field.

## 5 Summary and Outlook

**Figure 9:**
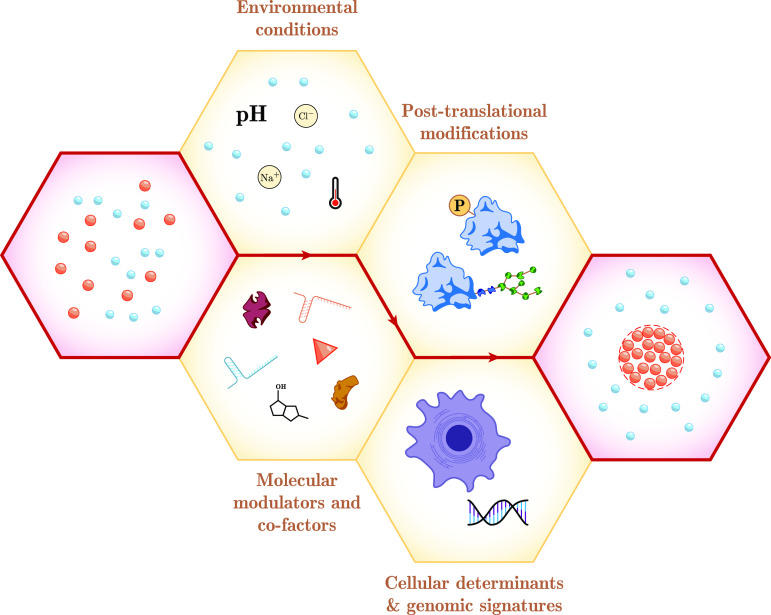
Computational approaches can be advanced further in multiple directions to better understand biomolecular phase separation processes. This includes but is not limited to understanding and elucidating (i) how environmental factors control phase separation, (ii) how the presence of other (bio)molecules, cofactors or other types of modulators affect the process, (iii) the role that post-translational modifications play in the process, and (iv) how cellular environment controls and enables phase separation and what the genomic signatures of biomolecular condensation are in cells.

We have reviewed recent developments in theoretical, physics-driven simulation and data-driven machine learning approaches to studying biomolecular condensation processes. We have outlined how all these approaches have helped to advance our understanding of the biomolecular condensation processes with each method presenting its unique advantages and limitations. Despite this notable progress, there is no shortage of questions where any one of the discussed computational approaches can have further impact. We highlight some potential directions in Figure 9.

Considering these open areas of research one-by-one, first, to date there have been relatively few attempts to predict how environmental conditions affect the phase separation process. This scarcity has been in part due to the low volumes of available data as few experimental methods are able to describe the effect of environmental conditions on phase separation in a high-throughput manner.^197−199^ While first machine learning models to this effect have been proposed,^191^ on the level of performance, physics-driven MD simulations have shown the most promise as they have recently demonstrated a remarkable accuracy in the recapitulation of phase diagrams for a number of protein sequences.^106^ However, it is worth highlighting that established MD methods considered only pairwise interactions between amino acid residues and are limited in their ability to capture cooperative interactions and three- and higher-body energy terms that are likely important for more complex multicomponent mixtures.

Next, the past few years have seen advancements in recapitulating the behavior of multicomponent systems. Physics-driven methods have moved in the direction of being able to accurately simulate systems more than a single component and theoretical approaches have advanced in the capability to model the key determinants of multivalent and cooperative interactions that give rise to phase separation. Machine learning models have not addressed the question of modeling multiphase systems in detail, but they have demonstrated the possibility to predict the components of MLOs in cells and discover hitherto unknown components of such systems. However, it is worth highlighting that there is no well-validated machine learning framework to predict the composition of distinct MLOs as most existing models concerning the predictions of the components of MLOs score proteins by propensities without allocating them to condensate systems.

An additional key direction that has been studied to a limited degree is the effect of post-translational modifications on biomolecular phase separation processes.^200−204^ A recent study by Sridharan et al. analyzed systematically how phosphorylation can affects the composition of MLOs and demonstrated a possible methodology for studying the effect of post-transnational modifications experimentally across a condensate system.^205^ With the advancement of experimental methods and condensate purification protocols, we expect larger volumes of data to emerge that characterizes how post-translational modifications affect protein phase behavior.

Finally, it is evident that, inside a cellular milieu, there is a vast number of factors that are involved in defining the biomolecular landscape of a cell. The same condensate systems may often be present in some cell lines but not all. Within the same cell line, a specific condensate system likely appears only under certain conditions. To the best of our knowledge, there are currently no approaches that have endeavored to describe in a systematic manner how the presence or absence of biomolecular condensates or their composition is defined by the cell state or cellular genomics.

To conclude, we envisage the variety of high-throughput experimental approaches that have been established over the past decade as part of cross-disciplinary teams across biological, chemical and physical sciences to be be systematically used and applied to examine biomolecular condensation processes. This process will likely lead to the emergence of ample experimental data around some of the open questions outlined in this Section. Efficient integration of experimental data and computational methods can help us to address outstanding research questions in an accelerated manner in order to generate a more complete picture of the molecular driving forces of the biomolecular condensation process and their biological role.

## References

[ref1] Alberti S.; Gladfelter A.; Mittag T. Considerations and challenges in studying liquid-liquid phase separation and biomolecular condensates. Cell 2019, 176, 419–434. 10.1016/j.cell.2018.12.035. 30682370 PMC6445271

[ref2] Mitrea D. M.; Kriwacki R. W. Phase separation in biology; functional organization of a higher order. Cell Commun. Signal. 2016, 14, 1–20. 10.1186/s12964-015-0125-7. 26727894 PMC4700675

[ref3] Feric M.; Vaidya N.; Harmon T. S.; Mitrea D. M.; Zhu L.; Richardson T. M.; Kriwacki R. W.; Pappu R. V.; Brangwynne C. P. Coexisting Liquid Phases Underlie Nucleolar Subcompartments. Cell 2016, 165, 1686–1697. 10.1016/j.cell.2016.04.047. 27212236 PMC5127388

[ref4] Lafontaine D. L. J.; Riback J. A.; Bascetin R.; Brangwynne C. P. The nucleolus as a multiphase liquid condensate. Nat. Rev. Mol. Cell Bio. 2021, 22, 165–182. 10.1038/s41580-020-0272-6. 32873929

[ref5] Protter D. S. W.; Parker R. Principles and Properties of Stress Granules. Trends Cell Biol. 2016, 26, 668–679. 10.1016/j.tcb.2016.05.004. 27289443 PMC4993645

[ref6] Anderson P.; Kedersha N.; Ivanov P. Stress granules, P-bodies and cancer. BBA-Gene Regul. Mech. 2015, 1849, 861–870. 10.1016/j.bbagrm.2014.11.009. PMC4457708 25482014

[ref7] Wei M.-T.; Chang Y.-C.; Shimobayashi S. F.; Shin Y.; Strom A. R.; Brangwynne C. P. Nucleated transcriptional condensates amplify gene expression. Nat. Cell Biol. 2020, 22, 1187–1196. 10.1038/s41556-020-00578-6. 32929202

[ref8] Lu Y.; Wu T.; Gutman O.; Lu H.; Zhou Q.; Henis Y. I.; Luo K. Phase separation of TAZ compartmentalizes the transcription machinery to promote gene expression. Nat. Cell Biol. 2020, 22, 453–464. 10.1038/s41556-020-0485-0. 32203417 PMC11044910

[ref9] Li P.; Banjade S.; Cheng H.-C.; Kim S.; Chen B.; Guo L.; Llaguno M.; Hollingsworth J. V.; King D. S.; Banani S. F.; Russo P. S.; Jiang Q.-X.; Nixon B. T.; Rosen M. K. Phase transitions in the assembly of multivalent signalling proteins. Nature 2012, 483, 336–340. 10.1038/nature10879. 22398450 PMC3343696

[ref10] Case L. B.; Ditlev J. A.; Rosen M. K. Regulation of Transmembrane Signaling by Phase Separation. Annu. Rev. Biophys. 2019, 48, 465–494. 10.1146/annurev-biophys-052118-115534. 30951647 PMC6771929

[ref11] Case L. B.; Zhang X.; Ditlev J. A.; Rosen M. K. Stoichiometry controls activity of phase-separated clusters of actin signaling proteins. Science 2019, 363, 1093–1097. 10.1126/science.aau6313. 30846599 PMC6784323

[ref12] Huang W. Y. C.; Alvarez S.; Kondo Y.; Lee Y. K.; Chung J. K.; Lam H. Y. M.; Biswas K. H.; Kuriyan J.; Groves J. T. A molecular assembly phase transition and kinetic proofreading modulate Ras activation by SOS. Science 2019, 363, 1098–1103. 10.1126/science.aau5721. 30846600 PMC6563836

[ref13] Aguzzi A.; Altmeyer M. Phase separation: linking cellular compartmentalization to disease. Trends Cell Biol. 2016, 26, 547–558. 10.1016/j.tcb.2016.03.004. 27051975

[ref14] Wolozin B.; Ivanov P. Stress granules and neurodegeneration. Nat. Rev. Neurosci. 2019, 20, 649–666. 10.1038/s41583-019-0222-5. 31582840 PMC6986315

[ref15] Mehta S.; Zhang J. Liquid–liquid phase separation drives cellular function and dysfunction in cancer. Nat. Rev. Cancer 2022, 22, 239–252. 10.1038/s41568-022-00444-7. 35149762 PMC10036213

[ref16] Hanwell M. D.; Curtis D. E.; Lonie D. C.; Vandermeersch T.; Zurek E.; Hutchison G. R. Avogadro: an advanced semantic chemical editor, visualization, and analysis platform. J. Cheminform. 2012, 4, 1–17. 10.1186/1758-2946-4-17. 22889332 PMC3542060

[ref17] Gkeka P.; Stoltz G.; Barati Farimani A.; Belkacemi Z.; Ceriotti M.; Chodera J. D.; Dinner A. R.; Ferguson A. L.; Maillet J.-B.; Minoux H.; Peter C.; Pietrucci F.; Silveira A.; Tkatchenko A.; Trstanova Z.; Wiewiora R.; Lelievre T. Machine learning force fields and coarse-grained variables in molecular dynamics: application to materials and biological systems. J. Chem. Theory Comp. 2020, 16, 4757–4775. 10.1021/acs.jctc.0c00355. PMC8312194 32559068

[ref18] Noé F. Machine learning meets quantum physics; Springer, 2020; pp 331–372.

[ref19] Riniker S. Molecular dynamics fingerprints (MDFP): machine learning from MD data to predict free-energy differences. J. Chem. Inf. Model. 2017, 57, 726–741. 10.1021/acs.jcim.6b00778. 28368113

[ref20] Reker D.; Schneider G. Active-learning strategies in computer-assisted drug discovery. Drug Discovery Today 2015, 20, 458–465. 10.1016/j.drudis.2014.12.004. 25499665

[ref21] Lookman T.; Balachandran P. V.; Xue D.; Yuan R. Active learning in materials science with emphasis on adaptive sampling using uncertainties for targeted design. npj Comp. Mater. 2019, 5, 1–17. 10.1038/s41524-019-0153-8.

[ref22] Wang R. Active Learning-Based Optimization of Scientific Experimental Design. arXiv 2021, 10.48550/arXiv.2112.14811.

[ref23] Torquato S.; Truskett T. M.; Debenedetti P. G. Is random close packing of spheres well defined?. Phys. Rev. Lett. 2000, 84, 2064–2067. 10.1103/PhysRevLett.84.2064. 11017210

[ref24] Pfeuty P. The one-dimensional Ising model with a transverse field. Ann. Phys. - New York 1970, 57, 79–90. 10.1016/0003-4916(70)90270-8.

[ref25] Yang C. N. The Spontaneous Magnetization of a Two-Dimensional Ising Model. Phys. Rev. 1952, 85, 808–816. 10.1103/PhysRev.85.808.

[ref26] Bardeen J.; Cooper L. N.; Schrieffer J. R. Theory of Superconductivity. Phys. Rev. 1957, 108, 1175–1204. 10.1103/PhysRev.108.1175.

[ref27] Gibbs J. W. On the equilibrium of heterogeneous substances. Am. J. Sci. 1874, 16, 441 10.2475/ajs.s3-16.96.441.

[ref28] Lin Y.-H.; Wessén J.; Pal T.; Das S.; Chan H. S. Phase-Separated Biomolecular Condensates: Methods and Protocols; Springer, 2022; pp 51–94.

[ref29] Qian D.; Michaels T. C.; Knowles T. P. Analytical Solution to the Flory–Huggins Model. J. Phys. Chem. Lett. 2022, 13, 7853–7860. 10.1021/acs.jpclett.2c01986. 35977086 PMC9421911

[ref30] Das R. K.; Pappu R. V. Conformations of intrinsically disordered proteins are influenced by linear sequence distributions of oppositely charged residues. P. Natl. Acad. Sci. USA 2013, 110, 13392–13397. 10.1073/pnas.1304749110. PMC3746876 23901099

[ref31] Flory P. J. Thermodynamics of High Polymer Solutions. J. Chem. Phys. 1942, 10, 51–61. 10.1063/1.1723621.

[ref32] Huggins M. L. Solutions of long chain compounds. J. Chem. Phys. 1941, 9, 440 10.1063/1.1750930.

[ref33] Tanaka F. Polymer Physics; Cambridge University Press, 2011; pp 46–69. 10.1017/CBO9780511975691.003

[ref34] Berry J.; Brangwynne C. P.; Haataja M. Physical principles of intracellular organization via active and passive phase transitions. Rep. Prog. Phys. 2018, 81, 046601 10.1088/1361-6633/aaa61e. 29313527

[ref35] Cahn J. W.; Hilliard J. E. Free Energy of a Nonuniform System. I. Interfacial Free Energy. J. Chem. Phys. 1958, 28, 258–267. 10.1063/1.1744102.

[ref36] Ariono D.; Aryanti P. T.; Hakim A. N.; Subagjo S.; Wenten I. G. Determination of thermodynamic properties of polysulfone/PEG membrane solutions based on Flory-Huggins model. AIP Conf. Proc. 2017, 1840, 090008.

[ref37] Mao S.; Kuldinow D.; Haataja M. P.; Košmrlj A. Phase behavior and morphology of multicomponent liquid mixtures. Soft Matter 2019, 15, 1297–1311. 10.1039/C8SM02045K. 30506078

[ref38] Qian D.; Welsh T. J.; Erkamp N. A.; Qamar S.; Nixon-Abell J.; Krainer G.; St. George-Hyslop P.; Michaels T. C. T.; Knowles T. P. J. Tie-lines reveal interactions driving heteromolecular condensate formation. bioRxiv 2022, 10.1103/PhysRevX.12.041038. PMC7619433 42694844

[ref39] Zwicker D.; Laan L. Evolved interactions stabilize many coexisting phases in multicomponent liquids. Proc. Natl. Acad. Sci. U.S.A. 2022, 119, 1–8. 10.1073/pnas.2201250119. PMC9282444 35867744

[ref40] Potters M.; Bouchaud J.-P. A First Course in Random Matrix Theory; Cambridge University Press, 2020; pp 15–29.

[ref41] Sear R. P.; Cuesta J. A. Instabilities in complex mixtures with a large number of components. Phys. Rev. Lett. 2003, 91, 2–5. 10.1103/PhysRevLett.91.245701. 14683134

[ref42] Ponomarenko E. A.; Poverennaya E. V.; Ilgisonis E. V.; Pyatnitskiy M. A.; Kopylov A. T.; Zgoda V. G.; Lisitsa A. V.; Archakov A. I. The Size of the Human Proteome: The Width and Depth. Int. J. Anal. Chem. 2016, 2016, 7436849 10.1155/2016/7436849. 27298622 PMC4889822

[ref43] Jacobs W. M.; Frenkel D. Phase Transitions in Biological Systems with Many Components. Biophys. J. 2017, 112, 683–691. 10.1016/j.bpj.2016.10.043. 28256228 PMC5340130

[ref44] Faghri A.; Zhang Y. Thermodynamics of Multiphase Systems; 2006; pp 107–176.

[ref45] Jacobs W. M.; Frenkel D. Phase transitions in biological systems with many components. Biophys. J. 2017, 112, 683–691. 10.1016/j.bpj.2016.10.043. 28256228 PMC5340130

[ref46] Shrinivas K.; Brenner M. P. Phase separation in fluids with many interacting components. Proc. Natl. Acad. Sci. U.S.A. 2021, 118, e2108551118 10.1073/pnas.2108551118. 34725154 PMC8609339

[ref47] Snead W. T.; Gladfelter A. S. The Control Centers of Biomolecular Phase Separation: How Membrane Surfaces, PTMs, and Active Processes Regulate Condensation. Mol. Cell 2019, 76, 295–305. 10.1016/j.molcel.2019.09.016. 31604601 PMC7173186

[ref48] Brangwynne C. P.; Tompa P.; Pappu R. V. Polymer physics of intracellular phase transitions. Nat. Phys. 2015, 11, 899–904. 10.1038/nphys3532.

[ref49] Shin Y.; Berry J.; Pannucci N.; Haataja M. P.; Toettcher J. E.; Brangwynne C. P. Spatiotemporal Control of Intracellular Phase Transitions Using Light-Activated optoDroplets. Cell 2017, 168, 159–171. 10.1016/j.cell.2016.11.054. 28041848 PMC5562165

[ref50] Reed E. H.; Schuster B. S.; Good M. C.; Hammer D. A. SPLIT: Stable Protein Coacervation Using a Light Induced Transition. ACS Synth. Biol. 2020, 9, 500–507. 10.1021/acssynbio.9b00503. 32078766 PMC7383129

[ref51] Cahn J. W. Phase separation by spinodal decomposition in isotropic systems. J. Chem. Phys. 1965, 42, 93–99. 10.1063/1.1695731.

[ref52] Ouazan-Reboul V.; Agudo-Canalejo J.; Golestanian R. Non-equilibrium phase separation in mixtures of catalytically active particles: size dispersity and screening effects. Eur. Phys. J. E 2021, 44, 1–10. 10.1140/epje/s10189-021-00118-6. 34478002 PMC8416889

[ref53] Longo T. J.; Anisimov M. A. Phase transitions affected by natural and forceful molecular interconversion. J. Chem. Phys. 2022, 156, 084502 10.1063/5.0081180. 35232197

[ref54] Bauermann J.; Laha S.; McCall P. M.; Jülicher F.; Weber C. A. Chemical Kinetics and Mass Action in Coexisting Phases. J. Am. Chem. Soc. 2022, 144, 19294–19304. 10.1021/jacs.2c06265. 36241174 PMC9620980

[ref55] Onsager L. Reciprocal Relations in Irreversible Processes. II. Phys. Rev. 1931, 38, 2265–2279. 10.1103/PhysRev.38.2265.

[ref56] Onsager L. Reciprocal Relations in Irreversible Processes. I. Phys. Rev. 1931, 37, 405–426. 10.1103/PhysRev.37.405.

[ref57] Guilhas B.; Walter J. C.; Rech J.; David G.; Walliser N. O.; Palmeri J.; Mathieu-Demaziere C.; Parmeggiani A.; Bouet J. Y.; Le Gall A.; Nollmann M. ATP-Driven Separation of Liquid Phase Condensates in Bacteria. Mol. Cell 2020, 79, 293–303. 10.1016/j.molcel.2020.06.034. 32679076

[ref58] Cates M. E.; Tailleur J. Motility-induced phase separation. Annu. Rev. Conden. Ma. P. 2015, 6, 219–244. 10.1146/annurev-conmatphys-031214-014710.

[ref59] Zwicker D.; Seyboldt R.; Weber C. A.; Hyman A. A.; Jülicher F. Growth and division of active droplets provides a model for protocells. Nat. Phys. 2017, 13, 408–413. 10.1038/nphys3984.

[ref60] Wittkowski R.; Tiribocchi A.; Stenhammar J.; Allen R. J.; Marenduzzo D.; Cates M. E. Scalar ϕ 4 field theory for active-particle phase separation. Nat. Commun. 2014, 5, 1–9. 10.1038/ncomms5351. 25008346

[ref61] Overbeek J. T. G.; Voorn M. J. Phase separation in polyelectrolyte solutions. Theory of complex coacervation. J. Cell. Compar. Physl. 1957, 49, 7–26. 10.1002/jcp.1030490404. 13449108

[ref62] Debye P.; Hückel E. Zur Theorie der Elektrolyte. I. Gefrierpunktserniedrigung und verwandte Erscheinungen. Phys. Z. 1923, 24, 185.

[ref63] Li L.; Srivastava S.; Andreev M.; Marciel A. B.; De Pablo J. J.; Tirrell M. V. Phase Behavior and Salt Partitioning in Polyelectrolyte Complex Coacervates. Macromolecules 2018, 51, 2988–2995. 10.1021/acs.macromol.8b00238.

[ref64] Dobrynin A. V.; Rubinstein M. Flory Theory of a Polyampholyte Chain. J. Phys.-Paris II 1995, 5, 677–695. 10.1051/jp2:1995157.

[ref65] Bremer A.; Farag M.; Borcherds W. M.; Peran I.; Martin E. W.; Pappu R. V.; Mittag T. Deciphering how naturally occurring sequence features impact the phase behaviours of disordered prion-like domains. Nat. Chem. 2022, 14, 196–207. 10.1038/s41557-021-00840-w. 34931046 PMC8818026

[ref66] Martin E. W.; Holehouse A. S.; Peran I.; Farag M.; Incicco J. J.; Bremer A.; Grace C. R.; Soranno A.; Pappu R. V.; Mittag T. Valence and patterning of aromatic residues determine the phase behavior of prion-like domains. Science 2020, 367, 694–699. 10.1126/science.aaw8653. 32029630 PMC7297187

[ref67] Krainer G.; Welsh T. J.; Joseph J. A.; St George-Hyslop P.; Hyman A. A.; Collepardo-Guevara R.; Alberti S.; Knowles T. P. Reentrant Liquid Condensate Phase of Proteins is Stabilized by Hydrophobic and Non-Ionic interactions. Biophys. J. 2021, 120, 28a 10.1016/j.bpj.2020.11.426. PMC7889641 33597515

[ref68] Semenov A. N.; Rubinstein M. Thermoreversible gelation in solutions of associative polymers. 1. Statics. Macromolecules 1998, 31, 1373–1385. 10.1021/ma970616h.

[ref69] Harmon T. S.; Holehouse A. S.; Rosen M. K.; Pappu R. V. Intrinsically disordered linkers determine the interplay between phase separation and gelation in multivalent proteins. eLife 2017, 6, 1–31. 10.7554/eLife.30294. PMC5703641 29091028

[ref70] De Gennes P.-G. Scaling concepts in polymer physics; Cornell University Press, 1979.

[ref71] Kar M.; Dar F.; Welsh T. J.; Vogel L. T.; Kuhnemuth R.; Majumdar A.; Krainer G.; Franzmann T. M.; Alberti S.; Seidel C. A. M.; Knowles T. P. J.; Hyman A. A.; Pappu R. V. Phase-separating RNA-binding proteins form heterogeneous distributions of clusters in subsaturated solutions. Proc. Natl. Acad. Sci. U.S.A. 2022, 119, e2202222119 10.1073/pnas.2202222119. 35787038 PMC9282234

[ref72] McCarty J.; Delaney K. T.; Danielsen S. P.; Fredrickson G. H.; Shea J. E. Complete Phase Diagram for Liquid-Liquid Phase Separation of Intrinsically Disordered Proteins. J. Phys. Chem. Lett. 2019, 10, 1644–1652. 10.1021/acs.jpclett.9b00099. 30873835 PMC7379843

[ref73] Rumyantsev A. M.; Jackson N. E.; Yu B.; Ting J. M.; Chen W.; Tirrell M. V.; De Pablo J. J. Controlling Complex Coacervation via Random Polyelectrolyte Sequences. ACS Macro Lett. 2019, 8, 1296–1302. 10.1021/acsmacrolett.9b00494. 35651159

[ref74] Statt A.; Casademunt H.; Brangwynne C. P.; Panagiotopoulos A. Z. Model for disordered proteins with strongly sequence-dependent liquid phase behavior. J. Chem. Phys. 2020, 152, 075101 10.1063/1.5141095. 32087632

[ref75] Pak C. W.; Kosno M.; Holehouse A. S.; Padrick S. B.; Mittal A.; Ali R.; Yunus A. A.; Liu D. R.; Pappu R. V.; Rosen M. K. Sequence Determinants of Intracellular Phase Separation by Complex Coacervation of a Disordered Protein. Mol. Cell 2016, 63, 72–85. 10.1016/j.molcel.2016.05.042. 27392146 PMC4973464

[ref76] Das S.; Amin A. N.; Lin Y. H.; Chan H. S. Coarse-grained residue-based models of disordered protein condensates: Utility and limitations of simple charge pattern parameters. Pgys. Chem. Chem. Phys. 2018, 20, 28558–28574. 10.1039/C8CP05095C. 30397688

[ref77] Sawle L.; Ghosh K. A theoretical method to compute sequence dependent configurational properties in charged polymers and proteins. J. Chem. Phys. 2015, 143, 085101 10.1063/1.4929391. 26328871

[ref78] Amin A. N.; Lin Y. H.; Das S.; Chan H. S. Analytical Theory for Sequence-Specific Binary Fuzzy Complexes of Charged Intrinsically Disordered Proteins. J. Phys. Chem. B 2020, 124, 6709–6720. 10.1021/acs.jpcb.0c04575. 32639157

[ref79] Lin Y. H.; Chan H. S. Phase Separation and Single-Chain Compactness of Charged Disordered Proteins Are Strongly Correlated. Biophys. J. 2017, 112, 2043–2046. 10.1016/j.bpj.2017.04.021. 28483149 PMC5448239

[ref80] Lytle T. K.; Sing C. E. Transfer matrix theory of polymer complex coacervation. Soft Matter 2017, 13, 7001–7012. 10.1039/C7SM01080J. 28840212

[ref81] Hubbard J. Calculation of partition functions. Phys. Rev. Lett. 1959, 3, 77–78. 10.1103/PhysRevLett.3.77.

[ref82] Stratonovich R. On a method of calculating quantum distribution functions. Sov. Phys. Doklady 1957, 2, 416.

[ref83] Lin Y. H.; Forman-Kay J. D.; Chan H. S. Sequence-Specific Polyampholyte Phase Separation in Membraneless Organelles. Phys. Rev. Lett. 2016, 117, 1–6. 10.1103/PhysRevLett.117.178101. 27824447

[ref84] Chen G. P.; Voora V. K.; Agee M. M.; Balasubramani S. G.; Furche F. Random-Phase Approximation Methods. Annu. Rev. Phys. Chem. 2017, 68, 421–445. 10.1146/annurev-physchem-040215-112308. 28301757

[ref85] Fredrickson G. The Equilibrium Theory of Inhomogeneous Polymers; Oxford University Press, 2005.

[ref86] Delaney K. T.; Fredrickson G. H. Recent Developments in Fully Fluctuating Field-Theoretic Simulations of Polymer Melts and Solutions. J. Phys. Chem. B 2016, 120, 7615–7634. 10.1021/acs.jpcb.6b05704. 27414265

[ref87] Lin Y. H.; Song J.; Forman-Kay J. D.; Chan H. S. Random-phase-approximation theory for sequence-dependent, biologically functional liquid-liquid phase separation of intrinsically disordered proteins. J. Mol. Liq. 2017, 228, 176–193. 10.1016/j.molliq.2016.09.090.

[ref88] Ngo V.; Da Silva M. C.; Kubillus M.; Li H.; Roux B.; Elstner M.; Cui Q.; Salahub D. R.; Noskov S. Y. Quantum Effects in Cation Interactions with First and Second Coordination Shell Ligands in Metalloproteins. J. Chem. Theory Comput. 2015, 11, 4992–5001. 10.1021/acs.jctc.5b00524. 26574284 PMC4827603

[ref89] Van Duin A. C.; Dasgupta S.; Lorant F.; Goddard W. A. ReaxFF: A reactive force field for hydrocarbons. J. Phys. Chem. A 2001, 105, 9396–9409. 10.1021/jp004368u.

[ref90] Unke O. T.; Chmiela S.; Sauceda H. E.; Gastegger M.; Poltavsky I.; Schutt K. T.; Tkatchenko A.; Muller K.-R. Machine learning force fields. Chem. Rev. 2021, 121, 10142–10186. 10.1021/acs.chemrev.0c01111. 33705118 PMC8391964

[ref91] Ponder J. W.; Case D. A. Force fields for protein simulations. Adv. Protein Chem. 2003, 66, 27–85. 10.1016/S0065-3233(03)66002-X. 14631816

[ref92] Best R. B.; Zheng W.; Mittal J. Balanced protein–water interactions improve properties of disordered proteins and non-specific protein association. J. Chem. Theory Comput. 2014, 10, 5113–5124. 10.1021/ct500569b. 25400522 PMC4230380

[ref93] Huang J.; Rauscher S.; Nawrocki G.; Ran T.; Feig M.; De Groot B. L.; Grubmüller H.; MacKerell A. D. CHARMM36m: an improved force field for folded and intrinsically disordered proteins. Nat. Methods 2017, 14, 71–73. 10.1038/nmeth.4067. 27819658 PMC5199616

[ref94] Robustelli P.; Piana S.; Shaw D. E. Developing a molecular dynamics force field for both folded and disordered protein states. Proc. Natl. Acad. Sci. U.S.A. 2018, 115, E4758–E4766. 10.1073/pnas.1800690115. 29735687 PMC6003505

[ref95] Piana S.; Robustelli P.; Tan D.; Chen S.; Shaw D. E. Development of a force field for the simulation of single-chain proteins and protein–protein complexes. J. Chem. Theory Comput. 2020, 16, 2494–2507. 10.1021/acs.jctc.9b00251. 31914313

[ref96] Piana S.; Lindorff-Larsen K.; Shaw D. E. How robust are protein folding simulations with respect to force field parameterization?. Biophys. J. 2011, 100, L47–L49. 10.1016/j.bpj.2011.03.051. 21539772 PMC3149239

[ref97] Rauscher S.; Pomès R. The liquid structure of elastin. eLife 2017, 6, e26526 10.7554/eLife.26526. 29120326 PMC5703643

[ref98] Zheng W.; Dignon G. L.; Jovic N.; Xu X.; Regy R. M.; Fawzi N. L.; Kim Y. C.; Best R. B.; Mittal J. Molecular details of protein condensates probed by microsecond long atomistic simulations. J. Phys. Chem. B 2020, 124, 11671–11679. 10.1021/acs.jpcb.0c10489. 33302617 PMC7879053

[ref99] Krainer G.; Welsh T. J.; Joseph J. A.; Espinosa J. R.; Wittmann S.; de Csillery E.; Sridhar A.; Toprakcioglu Z.; Gudiskyte G.; Czekalska M. A.; Arter W. E.; Guillen-Boixet J.; Franzmann T. M.; Qamar S.; George-Hyslop P. S.; Hyman A. A.; Collepardo-Guevara R.; Alberti S.; Knowles T. P. J. Reentrant liquid condensate phase of proteins is stabilized by hydrophobic and non-ionic interactions. Nat. Commun. 2021, 12, 1–14. 10.1038/s41467-021-21181-9. 33597515 PMC7889641

[ref100] Conicella A. E.; Dignon G. L.; Zerze G. H.; Schmidt H. B.; D’Ordine A. M.; Kim Y. C.; Rohatgi R.; Ayala Y. M.; Mittal J.; Fawzi N. L. TDP-43 α-helical structure tunes liquid–liquid phase separation and function. P. Natl. Acad. Sci. USA 2020, 117, 5883–5894. 10.1073/pnas.1912055117. PMC7084079 32132204

[ref101] De Sancho D. Phase separation in amino acid mixtures is governed by composition. Biophys. J. 2022, 121, 4119–4127. 10.1016/j.bpj.2022.09.031. 36181270 PMC9675019

[ref102] Tejedor A. R.; Sanchez-Burgos I.; Estevez-Espinosa M.; Garaizar A.; Collepardo-Guevara R.; Ramirez J.; Espinosa J. R. Protein structural transitions critically transform the network connectivity and viscoelasticity of RNA-binding protein condensates but RNA can prevent it. Nat. Commun. 2022, 13, 5717 10.1038/s41467-022-32874-0. 36175408 PMC9522849

[ref103] Garaizar A.; Espinosa J. R.; Joseph J. A.; Krainer G.; Shen Y.; Knowles T. P.; Collepardo-Guevara R. Aging can transform single-component protein condensates into multiphase architectures. Proc. Natl. Acad. Sci. U.S.A. 2022, 119, e2119800119 10.1073/pnas.2119800119. 35727989 PMC9245653

[ref104] Espinosa J. R.; Joseph J. A.; Sanchez-Burgos I.; Garaizar A.; Frenkel D.; Collepardo-Guevara R. Liquid network connectivity regulates the stability and composition of biomolecular condensates with many components. P. Natl. Acad. Sci. USA 2020, 117, 13238–13247. 10.1073/pnas.1917569117. PMC7306995 32482873

[ref105] Dignon G. L.; Zheng W.; Best R. B.; Kim Y. C.; Mittal J. Relation between single-molecule properties and phase behavior of intrinsically disordered proteins. P. Natl. Acad. Sci. USA 2018, 115, 9929–9934. 10.1073/pnas.1804177115. PMC6176625 30217894

[ref106] Joseph J. A.; Reinhardt A.; Aguirre A.; Chew P. Y.; Russell K. O.; Espinosa J. R.; Garaizar A.; Collepardo-Guevara R. Physics-driven coarse-grained model for biomolecular phase separation with near-quantitative accuracy. Nat. Comp. Sci. 2021, 1, 732–743. 10.1038/s43588-021-00155-3. PMC7612994 35795820

[ref107] Zerze G. H.; Zheng W.; Best R. B.; Mittal J. Evolution of All-Atom Protein Force Fields to Improve Local and Global Properties. J. Phys. Chem. Lett. 2019, 10, 2227–2234. 10.1021/acs.jpclett.9b00850. 30990694 PMC7507668

[ref108] Vitalis A.; Pappu R. V. ABSINTH: A new continuum solvation model for simulations of polypeptides in aqueous solutions. J. Comput. Chem. 2009, 30, 673–699. 10.1002/jcc.21005. 18506808 PMC2670230

[ref109] Lazaridis T.; Karplus M. Effective Energy Function for Proteins in Solution. Proteins 1999, 35, 133–152. 10.1002/(SICI)1097-0134(19990501)35:2<133::AID-PROT1>3.0.CO;2-N. 10223287

[ref110] Bottaro S.; Lindorff-Larsen K.; Best R. B. Variational optimization of an all-atom implicit solvent force field to match explicit solvent simulation data. J. Chem. Theory Comput. 2013, 9, 5641–5652. 10.1021/ct400730n. 24748852 PMC3987920

[ref111] Marrink S. J.; Risselada H. J.; Yefimov S.; Tieleman D. P.; De Vries A. H. The MARTINI force field: Coarse grained model for biomolecular simulations. J. Phys. Chem. B 2007, 111, 7812–7824. 10.1021/jp071097f. 17569554

[ref112] Dignon G. L.; Zheng W.; Kim Y. C.; Best R. B.; Mittal J. Sequence determinants of protein phase behavior from a coarse-grained model. PLOS Comp. Biol. 2018, 14, e1005941 10.1371/journal.pcbi.1005941. PMC5798848 29364893

[ref113] Souza P. C.; et al. Martini 3: a general purpose force field for coarse-grained molecular dynamics. Nat. Methods 2021, 18, 382–388. 10.1038/s41592-021-01098-3. 33782607 PMC12554258

[ref114] Stark A. C.; Andrews C. T.; Elcock A. H. Toward optimized potential functions for protein-protein interactions in aqueous solutions: Osmotic second virial coefficient calculations using the MARTINI coarse-grained force field. J. Chem. Theory Comput. 2013, 9, 4176–4185. 10.1021/ct400008p. PMC3819042 24223529

[ref115] Schmalhorst P. S.; Deluweit F.; Scherrers R.; Heisenberg C. P.; Sikora M. Overcoming the Limitations of the MARTINI Force Field in Simulations of Polysaccharides. J. Chem. Theory Comput. 2017, 13, 5039–5053. 10.1021/acs.jctc.7b00374. 28787166

[ref116] Javanainen M.; Martinez-Seara H.; Vattulainen I. Excessive aggregation of membrane proteins in the Martini model. PLoS One 2017, 12, e0187936 10.1371/journal.pone.0187936. 29131844 PMC5683612

[ref117] Benayad Z.; Von Bülow S.; Stelzl L. S.; Hummer G. Simulation of FUS Protein Condensates with an Adapted Coarse-Grained Model. J. Chem. Theory Comput. 2021, 17, 525–537. 10.1021/acs.jctc.0c01064. 33307683 PMC7872324

[ref118] Thomasen F. E.; Pesce F.; Roesgaard M. A.; Tesei G.; Lindorff-Larsen K. Improving Martini 3 for Disordered and Multidomain Proteins. J. Chem. Theory Comput. 2022, 18, 2033–2041. 10.1021/acs.jctc.1c01042. 35377637

[ref119] Dannenhoffer-Lafage T.; Best R. B. A data-driven hydrophobicity scale for predicting liquid–liquid phase separation of proteins. J. Phys. Chem. B 2021, 125, 4046–4056. 10.1021/acs.jpcb.0c11479. 33876938 PMC12442143

[ref120] Tesei G.; Lindorff-Larsen K. Improved Predictions of Phase Behaviour of Intrinsically Disordered Proteins by Tuning the Interaction Range. Open Res. Eur. 2022, 2, 94 10.12688/openreseurope.14967.1. PMC10450847 37645312

[ref121] Das S.; Lin Y.-H.; Vernon R. M.; Forman-Kay J. D.; Chan H. S. Comparative roles of charge, π, and hydrophobic interactions in sequence-dependent phase separation of intrinsically disordered proteins. P. Natl. Acad. Sci. USA 2020, 117, 28795–28805. 10.1073/pnas.2008122117. PMC7682375 33139563

[ref122] Latham A. P.; Zhang B. Consistent Force Field Captures Homologue-Resolved HP1 Phase Separation. J. Chem. Theory Comput. 2021, 17, 3134–3144. 10.1021/acs.jctc.0c01220. 33826337 PMC8119372

[ref123] Regy R. M.; Thompson J.; Kim Y. C.; Mittal J. Improved coarse-grained model for studying sequence dependent phase separation of disordered proteins. Protein Sci. 2021, 30, 1371–1379. 10.1002/pro.4094. 33934416 PMC8197430

[ref124] Murthy A. C.; Dignon G. L.; Kan Y.; Zerze G. H.; Parekh S. H.; Mittal J.; Fawzi N. L. Molecular interactions underlying liquid-liquid phase separation of the FUS low-complexity domain. Nat. Struct. Mol. Biol. 2019, 26, 637–648. 10.1038/s41594-019-0250-x. 31270472 PMC6613800

[ref125] Lebold K. M.; Best R. B. Tuning Formation of Protein-DNA Coacervates by Sequence and Environment. J. Phys. Chem. B 2022, 126, 2407–2419. 10.1021/acs.jpcb.2c00424. 35317553 PMC12442761

[ref126] Leicher R.; Osunsade A.; Chua G. N.; Faulkner S. C.; Latham A. P.; Watters J. W.; Nguyen T.; Beckwitt E. C.; Christodoulou-Rubalcava S.; Young P. G.; Zhang B.; David Y.; Liu S. Single-stranded nucleic acid binding and coacervation by linker histone H1. Nat. Struct. & Mol. Biol. 2022, 29, 463–471. 10.1038/s41594-022-00760-4. 35484234 PMC9117509

[ref127] Farr S. E.; Woods E. J.; Joseph J. A.; Garaizar A.; Collepardo-Guevara R. Nucleosome plasticity is a critical element of chromatin liquid–liquid phase separation and multivalent nucleosome interactions. Nat. Commun. 2021, 12, 1–17. 10.1038/s41467-021-23090-3. 34001913 PMC8129070

[ref128] Choi J. M.; Dar F.; Pappu R. V. LASSI: A lattice model for simulating phase transitions of multivalent proteins. PLOS Comp. Biol. 2019, 15, e1007028 10.1371/journal.pcbi.1007028. PMC6822780 31634364

[ref129] Borcherds W.; Bremer A.; Borgia M. B.; Mittag T. How do intrinsically disordered protein regions encode a driving force for liquid–liquid phase separation?. Curr. Opin. Struc. Biol. 2021, 67, 41–50. 10.1016/j.sbi.2020.09.004. PMC8044266 33069007

[ref130] Statt A.; Casademunt H.; Brangwynne C. P.; Panagiotopoulos A. Z. Model for disordered proteins with strongly sequence-dependent liquid phase behavior. J. Chem. Phys. 2020, 152, 075101 10.1063/1.5141095. 32087632

[ref131] Rana U.; Brangwynne C. P.; Panagiotopoulos A. Z. Phase separation vs aggregation behavior for model disordered proteins. J. Chem. Phys. 2021, 155, 125101 10.1063/5.0060046. 34598580

[ref132] Sanchez-Burgos I.; Espinosa J. R.; Joseph J. A.; Collepardo-Guevara R. Valency and Binding Affinity Variations Can Regulate the Multilayered Organization of Protein Condensates with Many Components. Biomol 2021, 11, 278 10.3390/biom11020278. PMC7918469 33672806

[ref133] Sanchez-Burgos I.; Joseph J. A.; Collepardo-Guevara R.; Espinosa J. R. Size conservation emerges spontaneously in biomolecular condensates formed by scaffolds and surfactant clients. Sci. Rep. 2021, 11, 1–10. 10.1038/s41598-021-94309-y. 34315935 PMC8316449

[ref134] Ranganathan S.; Shakhnovich E. I. Dynamic metastable long-living droplets formed by sticker-spacer proteins. eLife 2020, 9, 1–25. 10.7554/eLife.56159. PMC7360371 32484438

[ref135] Nguemaha V.; Zhou H. X. Liquid-Liquid Phase Separation of Patchy Particles Illuminates Diverse Effects of Regulatory Components on Protein Droplet Formation. Sci. Rep. 2018, 8, 1–11. 10.1038/s41598-018-25132-1. 29712961 PMC5928213

[ref136] Schmit J. D.; Feric M.; Dundr M. How hierarchical interactions make membraneless organelles tick like clockwork. Trends Biochem. Sci. 2021, 46, 525–534. 10.1016/j.tibs.2020.12.011. 33483232 PMC8195823

[ref137] Kota D.; Zhou H.-X. Macromolecular Regulation of the Material Properties of Biomolecular Condensates. J. Phys. Chem. Lett. 2022, 13, 5285–5290. 10.1021/acs.jpclett.2c00824. PMC9729394 35674796

[ref138] Joseph J. A.; Espinosa J. R.; Sanchez-Burgos I.; Garaizar A.; Frenkel D.; Collepardo-Guevara R. Thermodynamics and kinetics of phase separation of protein-RNA mixtures by a minimal model. Biophys. J. 2021, 120, 1219–1230. 10.1016/j.bpj.2021.01.031. 33571491 PMC8059209

[ref139] Hansen J.-P.; Addison C. I.; Louis A. A. Polymer solutions: from hard monomers to soft polymers. J. Phys.-Condens. Mater. 2005, 17, S3185 10.1088/0953-8984/17/45/001.

[ref140] Harker-Kirschneck L.; Hafner A. E.; Yao T.; Vanhille-Campos C.; Jiang X.; Pulschen A.; Hurtig F.; Hryniuk D.; Culley S.; Henriques R.; Baum B.; Saric A.đe. Physical mechanisms of ESCRT-III–driven cell division. Proc. Natl. Acad. Sci. U.S.A. 2022, 119, e2107763119 10.1073/pnas.2107763119. 34983838 PMC8740586

[ref141] Meadowcroft B.; Palaia I.; Pfitzner A.-K.; Roux A.; Baum B.; Šarić A. Mechanochemical rules for shape-shifting filaments that remodel membranes. Phys. Rev. Lett. 2022, 129, 268101 10.1103/PhysRevLett.129.268101. 36608212

[ref142] Hafner A. E.; Gyori N. G.; Bench C. A.; Davis L. K.; Šarić A. Modeling Fibrillogenesis of Collagen-Mimetic Molecules. Biophys. J. 2020, 119, 1791–1799. 10.1016/j.bpj.2020.09.013. 33049216 PMC7677131

[ref143] Grunewald F.; Punt M. H.; Jefferys E. E.; Vainikka P. A.; Konig M.; Virtanen V.; Meyer T. A.; Pezeshkian W.; Gormley A. J.; Karonen M.; Sansom M. S. P.; Souza P. C. T.; Marrink S. J. Martini 3 Coarse-Grained Force Field for Carbohydrates. J. Chem. Theory Comput. 2022, 18, 7555–7569. 10.1021/acs.jctc.2c00757. 36342474 PMC9753587

[ref144] Garaizar A.; Sanchez-Burgos I.; Collepardo-Guevara R.; Espinosa J. R. Expansion of Intrinsically Disordered Proteins Increases the Range of Stability of Liquid–Liquid Phase Separation. Molecules 2020, 25, 4705 10.3390/molecules25204705. 33076213 PMC7587599

[ref145] Webb S. Deep learning for biology. Nature 2018, 554, 555–558. 10.1038/d41586-018-02174-z. 29469107

[ref146] Rost B.; Sander C. Jury returns on structure prediction. Nature 1992, 360, 540 10.1038/360540b0. 1281284

[ref147] Rost B.; Sander C. Improved prediction of protein secondary structure by use of sequence profiles and neural networks. P. Natl. Acad. Sci. USA 1993, 90, 7558–7562. 10.1073/pnas.90.16.7558. PMC47181 8356056

[ref148] Schelling M.; Hopf T. A.; Rost B. Evolutionary couplings and sequence variation effect predict protein binding sites. Proteins 2018, 86, 1064–1074. 10.1002/prot.25585. 30020551

[ref149] Almagro Armenteros J. J.; Sønderby C. K.; Sønderby S. K.; Nielsen H.; Winther O. DeepLoc: prediction of protein subcellular localization using deep learning. Bioinformatics 2017, 33, 3387–3395. 10.1093/bioinformatics/btx431. 29036616

[ref150] Luo Y.; Jiang G.; Yu T.; Liu Y.; Vo L.; Ding H.; Su Y.; Qian W. W.; Zhao H.; Peng J. ECNet is an evolutionary context-integrated deep learning framework for protein engineering. Nat. Commun. 2021, 12, 1–14. 10.1038/s41467-021-25976-8. 34593817 PMC8484459

[ref151] Jumper J.; Evans R.; Pritzel A.; Green T.; Figurnov M.; Ronneberger O.; Tunyasuvunakool K.; Bates R.; Zidek A.; Potapenko A.; Bridgland A.; Meyer C.; Kohl S. A. A.; Ballard A. J.; Cowie A.; Romera-Paredes B.; Nikolov S.; Jain R.; Adler J.; Back T.; Petersen S.; Reiman D.; Clancy E.; Zielinski M.; Steinegger M.; Pacholska M.; Berghammer T.; Bodenstein S.; Silver D.; Vinyals O.; Senior A. W.; Kavukcuoglu K.; Kohli P.; Hassabis D. Highly accurate protein structure prediction with AlphaFold. Nature 2021, 596, 583–589. 10.1038/s41586-021-03819-2. 34265844 PMC8371605

[ref152] Kryshtafovych A.; Schwede T.; Topf M.; Fidelis K.; Moult J. Critical assessment of methods of protein structure prediction (CASP)—Round XIII. Proteins 2019, 87, 1011–1020. 10.1002/prot.25823. 31589781 PMC6927249

[ref153] Kryshtafovych A.; Schwede T.; Topf M.; Fidelis K.; Moult J. Critical assessment of methods of protein structure prediction (CASP)—Round XIV. Proteins 2021, 89, 1607–1617. 10.1002/prot.26237. 34533838 PMC8726744

[ref154] Baek M.; DiMaio F.; Anishchenko I.; Dauparas J.; Ovchinnikov S.; Lee G. R.; Wang J.; Cong Q.; Kinch L. N.; Schaeffer R. D.; Millan C.; Park H.; Adams C.; Glassman C. R.; DeGiovanni A.; Pereira J. H.; Rodrigues A. V.; van Dijk A. A.; Ebrecht A. C.; Opperman D. J.; Sagmeister T.; Buhlheller C.; Pavkov-Keller T.; Rathinaswamy M. K.; Dalwadi U.; Yip C. K.; Burke J. E.; Garcia K. C.; Grishin N. V.; Adams P. D.; Read R. J.; Baker D. Accurate prediction of protein structures and interactions using a three-track neural network. Science 2021, 373, 871–876. 10.1126/science.abj8754. 34282049 PMC7612213

[ref155] Ahdritz G.; et al. OpenFold: Retraining AlphaFold2 yields new insights into its learning mechanisms and capacity for generalization. bioRxiv 2022, 10.1101/2022.11.20.517210. PMC11645889 38744917

[ref156] Wu R.; et al. High-resolution de novo structure prediction from primary sequence. BioRxiv 2022, 10.1101/2022.07.21.500999.

[ref157] Lin Z.; et al. Evolutionary-scale prediction of atomic level protein structure with a language model. bioRxiv 2022, 10.1101/2022.07.20.500902. 36927031

[ref158] Detlefsen N. S.; Hauberg S.; Boomsma W. Learning meaningful representations of protein sequences. Nat. Commun. 2022, 13, 1–12. 10.1038/s41467-022-29443-w. 35395843 PMC8993921

[ref159] Elnaggar A.; Heinzinger M.; Dallago C.; Rehawi G.; Wang Y.; Jones L.; Gibbs T.; Feher T.; Angerer C.; Steinegger M.; Bhowmik D.; Rost B. Prottrans: Toward understanding the language of life through self-supervised learning. IEEE T Pattern Anal. Mach. Intell. 2022, 44, 7112–7127. 10.1109/TPAMI.2021.3095381. 34232869

[ref160] Asgari E.; Mofrad M. R. Continuous distributed representation of biological sequences for deep proteomics and genomics. PlOS One 2015, 10, e0141287 10.1371/journal.pone.0141287. 26555596 PMC4640716

[ref161] Alley E. C.; Khimulya G.; Biswas S.; AlQuraishi M.; Church G. M. Unified rational protein engineering with sequence-based deep representation learning. Nat. Methods 2019, 16, 1315–1322. 10.1038/s41592-019-0598-1. 31636460 PMC7067682

[ref162] Heinzinger M.; Elnaggar A.; Wang Y.; Dallago C.; Nechaev D.; Matthes F.; Rost B. Modeling aspects of the language of life through transfer-learning protein sequences. BMC Bioinf. 2019, 20, 1–17. 10.1186/s12859-019-3220-8. PMC6918593 31847804

[ref163] Rao R.; Bhattacharya N.; Thomas N.; Duan Y.; Chen P.; Canny J.; Abbeel P.; Song Y. Evaluating protein transfer learning with TAPE. Adv. Neural Inf. Process Syst. 2019, 32, 9689–9701. 33390682 PMC7774645

[ref164] Rives A.; Meier J.; Sercu T.; Goyal S.; Lin Z.; Liu J.; Guo D.; Ott M.; Zitnick C. L.; Ma J.; Fergus R. Biological structure and function emerge from scaling unsupervised learning to 250 million protein sequences. Proc. Natl. Acad. Sci. U.S.A. 2021, 118, e2016239118 10.1073/pnas.2016239118. 33876751 PMC8053943

[ref165] Bepler T.; Berger B. Learning the protein language: Evolution, structure, and function. Cell Syst 2021, 12, 654–669. 10.1016/j.cels.2021.05.017. 34139171 PMC8238390

[ref166] Bileschi M. L.; Belanger D.; Bryant D. H.; Sanderson T.; Carter B.; Sculley D.; Bateman A.; DePristo M. A.; Colwell L. J. Using deep learning to annotate the protein universe. Nat. Biotechnol. 2022, 40, 932 10.1038/s41587-021-01179-w. 35190689

[ref167] You K.; Huang Q.; Yu C.; Shen B.; Sevilla C.; Shi M.; Hermjakob H.; Chen Y.; Li T. PhaSepDB: a database of liquid–liquid phase separation related proteins. Nucleic Acids Res. 2020, 48, D354–D359. 10.1093/nar/gkz847. 31584089 PMC6943039

[ref168] Li Q.; Peng X.; Li Y.; Tang W.; Zhu J.; Huang J.; Qi Y.; Zhang Z. LLPSDB: a database of proteins undergoing liquid–liquid phase separation in vitro. Nucleic Acids Res. 2020, 48, D320–D327. 10.1093/nar/gkz778. 31906602 PMC6943074

[ref169] Wang X.; Zhou X.; Yan Q.; Liao S.; Tang W.; Xu P.; Gao Y.; Li Q.; Dou Z.; Yang W.; Huang B.; Li J.; Zhang Z. LLPSDB v2.0: an updated database of proteins undergoing liquid–liquid phase separation in vitro. Bioinformatics 2022, 38, 2010–2014. 10.1093/bioinformatics/btac026. 35025997 PMC8963276

[ref170] Mészáros B.; Erdos G.; Szabó B.; Schád É.; Tantos Á.; Abukhairan R.; Horváth T.; Murvai N.; Kovács O. P.; Kovács M.; Tosatto S. C.; Tompa P.; Dosztányi Z.; Pancsa R. PhaSePro: the database of proteins driving liquid–liquid phase separation. Nucleic Acids Res. 2019, 48, D360–D367. 10.1093/nar/gkz848. PMC7145634 31612960

[ref171] Ning W.; et al. DrLLPS: a data resource of liquid-liquid phase separation in eukaryotes. Nucleic Acids Res. 2020, 48, D288–D295. 10.1093/nar/gkz1027. 31691822 PMC7145660

[ref172] Youn J.-Y.; Dyakov B. J.; Zhang J.; Knight J. D.; Vernon R. M.; Forman-Kay J. D.; Gingras A.-C. Properties of stress granule and P-body proteomes. Mol. Cell 2019, 76, 286–294. 10.1016/j.molcel.2019.09.014. 31626750

[ref173] Dzuricky M.; Rogers B. A.; Shahid A.; Cremer P. S.; Chilkoti A. De novo engineering of intracellular condensates using artificial disordered proteins. Nat. Chem. 2020, 12, 814–825. 10.1038/s41557-020-0511-7. 32747754 PMC8281385

[ref174] Wang J.; Choi J.-M.; Holehouse A. S.; Lee H. O.; Zhang X.; Jahnel M.; Maharana S.; Lemaitre R.; Pozniakovsky A.; Drechsel D.; Poser I.; Pappu R. V.; Alberti S.; Hyman A. A. A molecular grammar governing the driving forces for phase separation of prion-like RNA binding proteins. Cell 2018, 174, 688–699. 10.1016/j.cell.2018.06.006. 29961577 PMC6063760

[ref175] Li H.-R.; Chiang W.-C.; Chou P.-C.; Wang W.-J.; Huang J.-r. TAR DNA-binding protein 43 (TDP-43) liquid–liquid phase separation is mediated by just a few aromatic residues. J. Biol. Chem. 2018, 293, 6090–6098. 10.1074/jbc.AC117.001037. 29511089 PMC5912450

[ref176] Elbaum-Garfinkle S.; Kim Y.; Szczepaniak K.; Chen C. C.-H.; Eckmann C. R.; Myong S.; Brangwynne C. P. The disordered P granule protein LAF-1 drives phase separation into droplets with tunable viscosity and dynamics. P. Natl. Acad. Sci. USA 2015, 112, 7189–7194. 10.1073/pnas.1504822112. PMC4466716 26015579

[ref177] Dao T. P.; Kolaitis R.-M.; Kim H. J.; O’Donovan K.; Martyniak B.; Colicino E.; Hehnly H.; Taylor J. P.; Castañeda C. A. Ubiquitin modulates liquid-liquid phase separation of UBQLN2 via disruption of multivalent interactions. Mol. Cell 2018, 69, 965–978. 10.1016/j.molcel.2018.02.004. 29526694 PMC6181577

[ref178] Carrettiero D. C.; Almeida M. C.; Longhini A. P.; Rauch J. N.; Han D.; Zhang X.; Najafi S.; Gestwicki J. E.; Kosik K. S. Stress routes clients to the proteasome via a BAG2 ubiquitin-independent degradation condensate. Nat. Commun. 2022, 13, 1–16. 10.1038/s41467-022-30751-4. 35654899 PMC9163039

[ref179] Yang P.; Mathieu C.; Kolaitis R.-M.; Zhang P.; Messing J.; Yurtsever U.; Yang Z.; Wu J.; Li Y.; Pan Q.; Yu J.; Martin E. W.; Mittag T.; Kim H. J.; Taylor J. P.; et al. G3BP1 is a tunable switch that triggers phase separation to assemble stress granules. Cell 2020, 181, 325–345. 10.1016/j.cell.2020.03.046. 32302571 PMC7448383

[ref180] Lancaster A. K.; Nutter-Upham A.; Lindquist S.; King O. D. PLAAC: a web and command-line application to identify proteins with prion-like amino acid composition. Bioinformatics 2014, 30, 2501–2502. 10.1093/bioinformatics/btu310. 24825614 PMC4147883

[ref181] Nott T. J.; Petsalaki E.; Farber P.; Jervis D.; Fussner E.; Plochowietz A.; Craggs T. D.; Bazett-Jones D. P.; Pawson T.; Forman-Kay J. D.; Baldwin A. J. Phase transition of a disordered nuage protein generates environmentally responsive membraneless organelles. Mol. Cell 2015, 57, 936–947. 10.1016/j.molcel.2015.01.013. 25747659 PMC4352761

[ref182] Bolognesi B.; Gotor N. L.; Dhar R.; Cirillo D.; Baldrighi M.; Tartaglia G. G.; Lehner B. A concentration-dependent liquid phase separation can cause toxicity upon increased protein expression. Cell Rep 2016, 16, 222–231. 10.1016/j.celrep.2016.05.076. 27320918 PMC4929146

[ref183] Vernon R. M.; Chong P. A.; Tsang B.; Kim T. H.; Bah A.; Farber P.; Lin H.; Forman-Kay J. D. Pi-Pi contacts are an overlooked protein feature relevant to phase separation. eLife 2018, 7, e31486 10.7554/eLife.31486. 29424691 PMC5847340

[ref184] Vernon R. M.; Forman-Kay J. D. First-generation predictors of biological protein phase separation. Curr. Opin. Struct. Biol. 2019, 58, 88–96. 10.1016/j.sbi.2019.05.016. 31252218

[ref185] Saar K. L.; Morgunov A. S.; Qi R.; Arter W. E.; Krainer G.; Lee A. A.; Knowles T. P. Learning the molecular grammar of protein condensates from sequence determinants and embeddings. Proc. Natl. Acad. Sci. U.S.A. 2021, 118, e2019053118 10.1073/pnas.2019053118. 33827920 PMC8053968

[ref186] van Mierlo G.; Jansen J. R.; Wang J.; Poser I.; van Heeringen S. J.; Vermeulen M. Predicting protein condensate formation using machine learning. Cell Rep 2021, 34, 108705 10.1016/j.celrep.2021.108705. 33535034

[ref187] Chu X.; Sun T.; Li Q.; Xu Y.; Zhang Z.; Lai L.; Pei J. Prediction of liquid–liquid phase separating proteins using machine learning. BMC Bioinform. 2022, 23, 1–13. 10.1186/s12859-022-04599-w. PMC8845408 35168563

[ref188] Cai H.; Vernon R. M.; Forman-Kay J. D. An interpretable machine-learning algorithm to predict disordered protein phase separation based on biophysical interactions. Biomolecules 2022, 12, 1131 10.3390/biom12081131. 36009025 PMC9405563

[ref189] Farahi N.; Lazar T.; Wodak S. J.; Tompa P.; Pancsa R. Integration of Data from Liquid–Liquid Phase Separation Databases Highlights Concentration and Dosage Sensitivity of LLPS Drivers. Int. J. Mol. Sci. 2021, 22, 3017 10.3390/ijms22063017. 33809541 PMC8002189

[ref190] Becker T.; Pich A.; Tamm S.; Hedtfeld S.; Ibrahim M.; Altmuller J.; Dalibor N.; Toliat M. R.; Janciauskiene S.; Tummler B.; Stanke F. Genetic information from discordant sibling pairs points to ESRP2 as a candidate trans-acting regulator of the CF modifier gene SCNN1B. Sci. Rep. 2020, 10, 1–19. 10.1038/s41598-020-79804-y. 33384439 PMC7775467

[ref191] Raimondi D.; Orlando G.; Michiels E.; Pakravan D.; Bratek-Skicki A.; Van Den Bosch L.; Moreau Y.; Rousseau F.; Schymkowitz J. In silico prediction of in vitro protein liquid–liquid phase separation experiments outcomes with multi-head neural attention. Bioinformatics 2021, 37, 3473–3479. 10.1093/bioinformatics/btab350. 33983381

[ref192] Zhu H.; Narita M.; Joseph J. A.; Krainer G.; Arter W. E.; Olan I.; Saar K. L.; Ermann N.; Espinosa J. R.; Shen Y.; Kuri M. A.; Qi R.; Welsh T. J.; Collepardo-Guevara R.; Narita M.; Knowles T. P. J. The chromatin regulator HMGA1a undergoes phase separation in the nucleus. ChemBioChem. 2023, 24, e202200450 10.1002/cbic.202200450. 36336658 PMC10098602

[ref193] Kuechler E. R.; Budzyńska P. M.; Bernardini J. P.; Gsponer J.; Mayor T. Distinct features of stress granule proteins predict localization in membraneless organelles. J. Mol. Biol. 2020, 432, 2349–2368. 10.1016/j.jmb.2020.02.020. 32105731

[ref194] Kuechler E. R.; Jacobson M.; Mayor T.; Gsponer J. GraPES: The Granule Protein Enrichment Server for prediction of biological condensate constituents. Nucleic Acids Res. 2022, 50, W384–W391. 10.1093/nar/gkac279. 35474477 PMC9252806

[ref195] Villanueva E.; et al. A system-wide quantitative map of RNA and protein subcellular localisation dynamics. bioRxiv 2022, 10.1101/2022.01.24.477541.

[ref196] Cho N. H.; Cheveralls K. C.; Brunner A.-D.; Kim K.; Michaelis A. C.; Raghavan P.; Kobayashi H.; Savy L.; Li J. Y.; Canaj H.; Kim J. Y. S.; Stewart E. M.; Gnann C.; McCarthy F.; Cabrera J. P.; Brunetti R. M.; Chhun B. B.; Dingle G.; Hein M. Y.; Huang B.; Mehta S. B.; Weissman J. S.; Gomez-Sjoberg R.; Itzhak D. N.; Royer L. A.; Mann M.; Leonetti M. D. OpenCell: Endogenous tagging for the cartography of human cellular organization. Science 2022, 375, eabi6983 10.1126/science.abi6983. 35271311 PMC9119736

[ref197] Arter W. E.; Qi R.; Erkamp N. A.; Krainer G.; Didi K.; Welsh T. J.; Acker J.; Nixon-Abell J.; Qamar S.; Guillen-Boixet J.; Franzmann T. M.; Kuster D.; Hyman A. A.; Borodavka A.; George-Hyslop P. S.; Alberti S.; Knowles T. P. J. Biomolecular condensate phase diagrams with a combinatorial microdroplet platform. Nat. Commun. 2022, 13, 7845 10.1038/s41467-022-35265-7. 36543777 PMC9768726

[ref198] Li P.; Zeng X.; Li S.; Xiang X.; Chen P.; Li Y.; Liu B.-F. Rapid Determination of Phase Diagrams for Biomolecular Liquid–Liquid Phase Separation with Microfluidics. Anal. Chem. 2022, 94, 687–694. 10.1021/acs.analchem.1c02700. 34936324

[ref199] Kopp M. R.; Linsenmeier M.; Hettich B.; Prantl S.; Stavrakis S.; Leroux J.-C.; Arosio P. Microfluidic shrinking droplet concentrator for analyte detection and phase separation of protein solutions. Anal. Chem. 2020, 92, 5803–5812. 10.1021/acs.analchem.9b05329. 32249573

[ref200] Owen I.; Shewmaker F. The role of post-translational modifications in the phase transitions of intrinsically disordered proteins. Int. J. Mol. Sci. 2019, 20, 5501 10.3390/ijms20215501. 31694155 PMC6861982

[ref201] Singh H. R.; Ostwal Y. B. Post-translational modification, phase separation, and robust gene transcription. Trends Genet 2019, 35, 89–92. 10.1016/j.tig.2018.11.002. 30477958

[ref202] Luo Y.-Y.; Wu J.-J.; Li Y.-M. Regulation of liquid–liquid phase separation with focus on post-translational modifications. Chem. Common. 2021, 57, 13275–13287. 10.1039/D1CC05266G. 34816836

[ref203] Chong P. A.; Forman-Kay J. D. Liquid–liquid phase separation in cellular signaling systems. Curr. Opin. Struc. Biol. 2016, 41, 180–186. 10.1016/j.sbi.2016.08.001. 27552079

[ref204] Bhandari K.; Cotten M. A.; Kim J.; Rosen M. K.; Schmit J. D. Structure–function properties in disordered condensates. J. Phys. Chem. B 2021, 125, 467–476. 10.1021/acs.jpcb.0c11057. 33395293 PMC8194388

[ref205] Sridharan S.; Hernandez-Armendariz A.; Kurzawa N.; Potel C. M.; Memon D.; Beltrao P.; Bantscheff M.; Huber W.; Cuylen-Haering S.; Savitski M. M. Systematic discovery of biomolecular condensate-specific protein phosphorylation. Nat. Chem. Biol. 2022, 18, 1104 10.1038/s41589-022-01062-y. 35864335 PMC9512703

